# Reliability and validity of the Patient Benefit Assessment Scale for Hospitalised Older Patients (P-BAS HOP)

**DOI:** 10.1186/s12877-021-02079-z

**Published:** 2021-03-01

**Authors:** Maria Johanna van der Kluit, Geke J. Dijkstra, Sophia E. de Rooij

**Affiliations:** 1 University of Groningen, University Medical Center Groningen, University Center for Geriatric Medicine, Hanzeplein 1, 9700 RB Groningen, The Netherlands; 2 University of Groningen, University Medical Center Groningen, Department of Health Sciences, Applied Health Research, Groningen, The Netherlands; 3 NHL Stenden University of Applied Sciences, Research Group Living, Wellbeing and Care for Older People, Leeuwarden, The Netherlands; 4 Medical Spectrum Twente, Medical School Twente, Enschede, The Netherlands

**Keywords:** Older adults, Hospitalisation, Patient perspective, Goal setting, Patient-reported outcomes, Validity, Reliability, Responsiveness, Minimal important change (MIC), Value-based health care

## Abstract

**Background:**

The Patient Benefit Assessment Scale for Hospitalised Older Patients (P-BAS HOP) is a tool which is capable of both identifying the priorities of the individual patient and measuring the outcomes relevant to him/her, resulting in a Patient Benefit Index (PBI) with range 0–3, indicating how much benefit the patient had experienced from the admission. The aim of this study was to evaluate the reliability, validity, responsiveness and interpretability of the P-BAS HOP.

**Methods:**

A longitudinal study among hospitalised older patients with a baseline interview during hospitalisation and a follow-up by telephone 3 months after discharge. Test-retest reliability of the baseline and follow-up questionnaire were tested. Percentage of agreement, Cohen’s kappa with quadratic weighting and maximum attainable kappa were calculated per item. The PBI was calculated for both test and retest of baseline and follow-up and compared with Intraclass Correlation Coefficient (ICC). Construct validity was tested by evaluating pre-defined hypotheses comparing the priority of goals with experienced symptoms or limitations at admission and the achievement of goals with progression or deterioration of other constructs. Responsiveness was evaluated by correlating the PBI with the anchor question ‘How much did you benefit from the admission?’. This question was also used to evaluate the interpretability of the PBI with the visual anchor-based minimal important change distribution method.

**Results:**

Reliability was tested with 53 participants at baseline and 72 at follow-up. Mean weighted kappa of the baseline items was 0.38. ICC between PBI of the test and retest was 0.77.

Mean weighted kappa of the follow-up items was 0.51. ICC between PBI of the test and retest was 0.62.

For the construct validity, tested in 451 participants, all baseline hypotheses were confirmed. From the follow-up hypotheses, tested in 344 participants, five of seven were confirmed.

The Spearman’s correlation coefficient between the PBI and the anchor question was 0.51.

The optimal cut-off point was 0.7 for ‘no important benefit’ and 1.4 points for ‘important benefit’ on the PBI.

**Conclusions:**

Although the concept seems promising, the reliability and validity of the P-BAS HOP appeared to be not yet satisfactory. We therefore recommend adapting the P-BAS HOP.

**Supplementary Information:**

The online version contains supplementary material available at 10.1186/s12877-021-02079-z.

## Background

Healthcare interventions are often evaluated in terms of survival or disease-specific measures, while for many older people more personal goals such as functional status, social functioning and relief of symptoms, which are considered important by the individual self, are prioritised [1, 2]. Furthermore, which outcomes are considered important differ per individual [1, 3]. When care is to be systematically evaluated by personal goal-oriented outcomes, a tool is needed which is capable of both identifying the priorities of the individual patient and measuring the outcomes relevant to him/her. We therefore developed the Patient Benefit Assessment Scale for Hospitalised Older Patients (P-BAS HOP) [4].

The P-BAS HOP is an interview-based tool consisting of two parts: 1) a baseline questionnaire to select and assess the importance of various predefined goals, based on subjects derived from qualitative interviews with hospitalised older patients and 2) an evaluation questionnaire to evaluate the extent to which the hospital admission helped to achieve these individual goals. Based on these data it is possible to compute an individual Patient Benefit Index. The comprehensibility, feasibility and a first indication of content validity were already tested in pilot test and field tests [4]. The aim of the present study is to evaluate the reliability, validity, responsiveness and interpretability of the P-BAS HOP.

## Methods

### Design and population

This longitudinal study was performed among hospitalised older patients. The first face-to-face interview took place within the first 4 days of hospitalisation. The follow-up interview was performed 3 months after discharge by telephone.

Eligible participants were 70 years and older; had either a planned or unplanned hospital admission on medical or surgical wards of a university teaching hospital in the Netherlands, had an expected hospital stay of at least 48 h; were able to speak and understand Dutch and were without cognitive impairment. Inclusion criteria were verified with the staff nurse. Patients were approached by a trained research assistant and gave signed informed consent.

### Questionnaire: P-BAS HOP

The P-BAS HOP is an interview-based questionnaire. The baseline questionnaire consists of two parts: in the first part the interviewer lists subjects and the participant can indicate whether experiencing or expecting limitations regarding that subject. In the second part, the participant is asked, for each subject identified in the first part whether it is a goal for the current hospitalisation and, if so, how important the goal is. Answer options are: does not apply to me; not at all important; somewhat important; quite important and very important.

At follow-up, the participant is asked per selected goal to what extent the hospitalisation helped to achieve that goal. The answer options are: not at all; somewhat; quite; completely.

With the scores of the baseline and follow-up questionnaire, a Patient Benefit Index (PBI) can be calculated: this is the mean of the benefits, weighted by the importance of the goals:

$$ PBI=\sum \limits_{i=1}^k\frac{G_i}{\sum \limits_{i=1}^k{G}_i}\ {B}_i $$

with k goal-items (*G*_*i*_)(range 0–3, related to answer options for importance) and benefit-items *B*_*i*_ (range 0–3, related to answer options for achievement of goals).

### Other questionnaires and constructs

For the construct validity the used questionnaires or constructs are summarised in Table 1. Full details are given in Additional file 1.

**Table 1:** Constructs measured for the construct validity

Construct	Operationalisation
Appetite	Dutch VMS screening program (VMS) [5]
Symptoms experienced on admission day	Rotterdam Symptom Checklist (RSCL) [6]
Pain, experienced at moment of interview	Numeric rating scale (NRS) pain (0: no pain at all to 10: the worst imaginable pain)
Fatigue, experienced at moment of interview	NRS fatigue (0: no fatigue at all to 10: the worst imaginable fatigue)
Health related quality of life 2 weeks before admission/ at moment of follow-up interview	EQ-5D [7]
Admission reason	Acute/ elective; diagnostic/ curative/ palliative
Activities of daily living 2 weeks before admission/ at moment of follow-up interview	KATZ-15 scale [8]
Social functioning	The Maastricht Social Participation Profile (MSPP) [9]; Or 36-Item Short Form Survey Instrument (SF-36) – Social functioning [10]
Goals on hospital admission	Open question: What do you hope to accomplish with this hospitalisation?

### Reliability

Test-retest reliability of the baseline questionnaire was performed with an interval of 1 to 3 days, while the participant was still hospitalised. The participant was not notified in advance of the retest, but asked for permission for another test on the other day. Then only the P-BAS HOP was repeated.

For a better understanding of the difference between test and retest, a short qualitative evaluation was done: a selection of seven participants were asked, after the retest, to explain what caused the discrepancies per item between test and retest.

Test-retest of the follow-up questionnaire was performed in another sample than the baseline test-retest with an interval of 7 to 14 days. At the end of the first follow-up interview, the participant was asked permission to be called back a week later to repeat some questions, without specifying which questions. Only the P-BAS HOP was repeated.

Percentage of agreement, Cohen’s Kappa with quadratic weighting and maximum attainable kappa [11, 12] were calculated per item for the agreement on importance of the goals on baseline, and the extent the hospitalisation helped to achieve the set goals on follow-up. Both the goal items ‘doesn’t apply to me’ and ‘not at all important’ were valued as zero. For all kappa calculations an online calculator was used [13]. For the interpretation of the kappa values, the classification of Landis and Koch [14] was used.

The PBI was calculated for both test and retest of baseline and follow-up and compared with Intraclass Correlation Coefficient (ICC).

### Validity

#### Baseline questionnaire

The hypotheses we developed to test the construct validity of the baseline questionnaire are listed in Table 2.

**Table 2:** Hypotheses baseline validity with expected and calculated correlations

	Hypothesis	Expected correlation	Calculated correlation		Confirmed (C)/ Rejected (R)
			n		
1	Participants who indicated a lack of appetite on the VMS and/or the RSCL, are expected to have a higher priority for the goal ‘appetite’.	Cramér’s V > 0.10	450	0.50	C
2	Participants who indicated tiredness and/or lack of energy on the RSCL, are expected to have a higher priority for the goal ‘energy’.	Cramér’s V > 0.10	442	0.34	C
3	Participants who indicated diarrhoea and/or constipation on the RSCL, are expected to have a higher priority for the goal ‘bowel movements’.	Cramér’s V > 0.10	441	0.40	C
4	Participants who indicated shortness of breath on the RSCL, are expected to have a higher priority for the goal ‘reducing shortness of breath’.	Cramér’s V > 0.10	440	0.60	C
5	Participants who had an acute admission and/or a diagnostic admission reason, are expected to have a higher priority for the goal ‘wanting to know what is wrong’.	Cramér’s V > 0.10	431	0.25	C
6	Participants with a higher NRS pain are expected to have a higher priority for the goal ‘pain’.	Spearman’s > 0.10	442	0.39	C
7	Participants with a higher score on the SF36-social functioning, are expected to have a higher priority for the goal ‘visiting family or friends’.	Spearman’s > 0.10	220	0.15	C
8	Goals that were mentioned after the open question, are, when applicable, indicated as minimum ‘somewhat important’ for the concerning goal.	Percentage of agreement ≥75%	50	75%	C

Hypotheses 1 to 5 were evaluated using Cramér’s V statistic. Hypotheses 6 and 7 were evaluated with the Spearman’s rank-order correlation. Since experiencing a symptom or restraint in a certain subject, does not necessarily mean that this goal is a priority for hospital admission, the hypotheses are confirmed if the correlation exceeds ‘small’ as defined by Cohen [15], meaning the correlation > 0.10. The answer option ‘does not apply to me now’ and ‘not at all important’ were coded as 0, the options somewhat important, quite important and very important were coded respectively as 1, 2, 3. Only when the assumptions of Cramér’s V statistic were not met because of too low (expected) cell frequency, categories were combined.

For hypothesis 8, a random selection of 50 cases was made and goals mentioned in the open question were coded using the item names of the P-BAS HOP. When a participant mentioned a goal that was not in the P-BAS HOP, it was coded as ‘other’. The coding was done by two researchers independently and then compared and discrepancies were solved by consensus. Subsequently, the percentage of agreement between the labels and the answers given in the P-BAS HOP was calculated.

The baseline questionnaire was considered valid if a minimum of 75%, thus six, of the first seven hypotheses were confirmed and hypothesis 8 was confirmed in a minimum of 75% of the selected cases [16].

#### Follow-up questionnaire

The extent to which the hospitalisation helped to achieve the set goals is compared with the progression or deterioration of items between baseline and follow-up from other known questionnaires. Hence the formulated hypotheses are listed in Table 3.:

**Table 3:** Hypotheses follow-up validity with expected and calculated correlations

	Hypothesis	Expected correlation	Calculated correlation		Confirmed (C)/ Rejected (R)
			n		
1	Participants who indicated a deterioration on the Katz-15 items bathing and/or getting dressed and/ or the EQ-5D item self-care, are expected to have a lower score on the item ‘wash and dress yourself’.	Cramér’s V > 0.10	33	nc	n.a.
2	Participants who indicated a deterioration on the Katz-15 item walking and/or the EQ-5D item mobility, are expected to have a lower score on the item ‘walking’.	Cramér’s V > 0.10	116	0.23^a^	R
3	Participants who indicated a deterioration on the Katz-15 item travelling, are expected to have a lower score on the item ‘driving’.	Cramér’s V > 0.10	37	nc	n.a.
4	Participants who indicated a deterioration on the Katz-15 item shopping, are expected to have a lower score on the item ‘groceries’.	Cramér’s V > 0.10	37	nc	n.a.
5	Participants who indicated a deterioration on the EQ-5D item pain/discomfort, are expected to have a lower score on the item ‘pain’.	Cramér’s V > 0.10	102	0.14^a^	R
6	Participants who indicated a lack of appetite on the VMS, are expected to have a lower score on the item ‘appetite’.	Cramér’s V > 0.10	45	0.46	C
7	Participants who indicated a deterioration on the MSPP item organised sports and/or the MSPP item ‘done something with others that required considerable physical effort’, are expected to have a lower score on the item ‘sports’.	Cramér’s V > 0.10	21	nc	n.a.
8	Participants who indicated a deterioration on the MSPP item seeing family/acquaintances or the SF36-social functioning, are expected to have a lower score on the item ‘visiting family or friends’.	Cramér’s V > 0.10	30	nc	n.a.
9	Participants who moved from independent living to sheltered living or a nursing home, are expected to score lower on the item ‘return back to my home’.	Cramér’s V > 0.10	11	nc	n.a.
10	Participants with an increasing difference score between baseline and follow-up on the EQ-5D thermometer ‘general health’, are expected to have a higher score on the item ‘feeling better’.	Spearman’s > 0.10	241	0.14	C
11	Participants with an increasing difference score between baseline and follow-up on the sum score ‘MSPP-daytrip’, are expected to have a higher score on the item ‘go on outings’.	Spearman’s > 0.10	33	0.27	C
12	Participants with an increasing difference score between baseline and follow-up on the NRS fatigue, are expected to have a lower score on the item energy.	Spearman’s < -0.10	189	−0.14	C
13	Accomplishing goals noted on the open question correlate with the score on the P-BAS HOP, if applicable.	Spearman’s > 0.50	40	0.71	C

^a^ association is opposite of the hypothesis; nc = not calculated; n.a. = not applicable

Hypotheses 1 to 9 were evaluated using Cramér’s V statistic. Hypotheses 10 to 12 were evaluated with the Spearman’s rank-order correlation. Since experiencing a progression or deterioration in a certain subject, does not necessarily mean that this is due to the hospital admission, the hypotheses are confirmed if the correlation exceeds ‘small’ as defined by Cohen [15], meaning the correlation > 0.10.

For hypothesis 13 the same records were used as for hypothesis 8 on baseline. For the dyads with agreement between the code for the open question and the P-BAS HOP item, the Spearman’s rank-order correlation between the answer on the open question and the corresponding P-BAS HOP item was calculated. The hypothesis was confirmed if the correlation > 0.50.

The follow-up questionnaire was considered valid if a minimum of 75% [16], thus nine of the first 12 hypotheses, were confirmed and hypothesis 13 was confirmed.

### Responsiveness

The following anchor question was used to validate the PBI: ‘How much have you benefited from the admission?’ With the following answer options: not at all, a little bit, somewhat, much, very much.

The PBI is considered valid when it has a Spearman’s correlation coefficient ≥ 0.50 with the anchor question [17, 18].

### Interpretability

The interpretability is evaluated with the visual anchor-based minimal important change distribution method [11, 18]. Participants who indicated: ‘not at all’, and ‘a little bit’, were considered as having no important benefit. Participants who indicated ‘very’ or ‘very much’, are considered as having important benefit. As it was not clear whether ‘somewhat benefit’ was considered as important benefit or not, we labelled this as ‘borderline’. The receiver operating characteristic (ROC) curve was used to determine the optimal cut-off points for important and no important benefit.

### Missing values

When the P-BAS HOP was not administered, the case was completely deleted. For all other missing values, we used pairwise deletion. The computation of the PBI was based on non-missing items.

## Results

### Sample

**Fig. 1:**
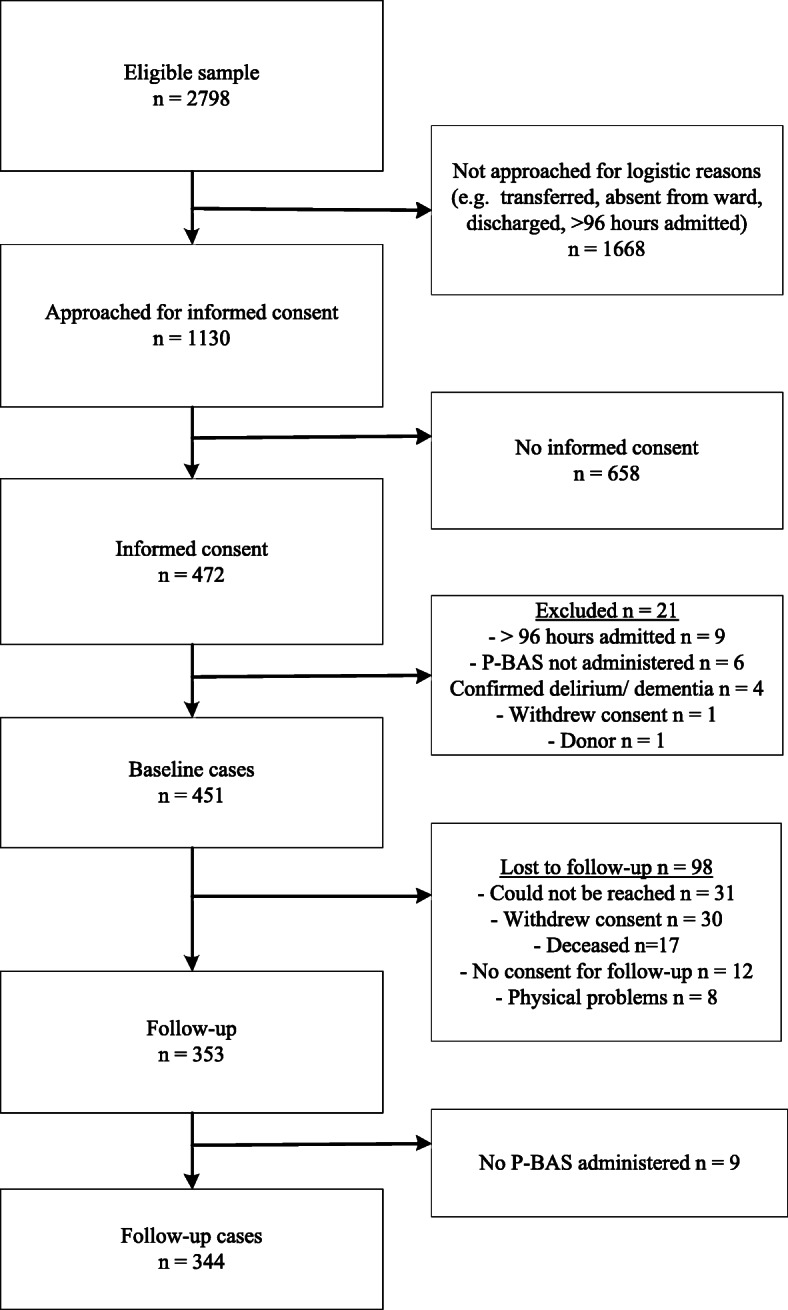
Flowchart participant inclusion

From the 2798 eligible patients, 1130 were approached for informed consent and 472 gave informed consent. After exclusion of 21 cases, we had 451 baseline cases. We lost 98 cases to follow-up and in an additional nine cases the P-BAS HOP was not administered at follow-up, which resulted in 344 follow-up cases. Full details are shown in Fig. 1. Most (43%) baseline interviews were done on the third day of admission.

Sample characteristics are shown in Table 4 and Additional File 2 shows the scores of the other questionnaires measured for the construct validity.

**Table 4:** Sample characteristics (*n* = 451)

Characteristic	n (%)
Gender	
Male	300 (67)
Female	151 (34)
Age (years), median (range)	76 (70–96)
Living situation	
Independent	432 (96)
Sheltered accommodation	14 (3)
Senior home	3 (1)
Nursing home	2 (0)
Educational level^a^	
Low	127 (28)
Middle	197 (44)
High	124 (28)
Unknown	3 (1)
Specialty	
Medical	191 (42)
Surgical	109 (24)
Intervention cardiology	136 (30)
Unknown	15 (3)
Admission type	
Acute	257 (57)
Elective	179 (40)
Unknown	15 (3)
Admission time (days) median (range)	5 (1–39)
Interview took place after number of days after admission (days)	
1	8 (2)
2	101 (22)
3	193 (43)
4	149 (33)

^a^ Educational level: Low = no education, primary school, prevocational education; Middle = secondary or vocational education; High = bachelor, master

### Descriptive statistics P-BAS HOP

Table 5 shows the baseline and follow-up descriptive statistics of the P-BAS HOP. The number of goals selected as minimum ‘somewhat important’ varied from zero to 17 per person, with a median of five. Eleven persons selected no goals from the P-BAS HOP. Nineteen participants mentioned an extra goal. Examples of an extra goal were: resuming work; giving informal care to a relative or partner; being able to swallow. The missing values at baseline are mostly due to the interviewer accidentally omitting a question; five times it was because the participant did not know the answer.

**Table 5:** P-BAS HOP Baseline and follow-up descriptive statistics

Item	Baseline						Follow-up					
		Importance						Achievement				
	Not applicable for me n (%)	Not at all n (%)	Some-what n (%)	Quite n (%)	Very n (%)	Mis sing (%)	Not applicable for me n (%)	Not at all n (%)	Some-what n (%)	Quite n (%)	Completely n (%)	Missing
Better	116 (25.7)	2 (0.4)	8 (1.8)	64 (14.2)	259 (57.4)	2 (0.4)	4 (1.6)	40 (15.6)	59 (23.0)	74 (28.8)	77 (30.0)	3 (1.2)
Energy	194 (43.0)	2 (0.4)	16 (3.5)	77 (17.1)	161 (35.7)	1 (0.2)	8 (4.0)	68 (33.8)	45 (22.4)	50 (24.9)	27 (13.4)	3 (1.5)
Pain	293 (65.0)	1 (0.2)	12 (2.7)	40 (8.9)	105 (23.3)	0	17 (14.2)	28 (23.3)	18 (15.0)	20 (16.7)	37 (30.8)	0
Bowel movements	381 (84.5)	1 (0.2)	5 (1.1)	21 (4.7)	41 (9.1)	2 (0.4)	10 (19.2)	11 (21.2)	3 (5.8)	8 (15.4)	17 (32.7)	3^a^ (5.8)
Shortness of breath	272 (60.3)	1 (0.2)	14 (3.1)	44 (9.8)	118 (26.2)	2 (0.4)	8 (6.1)	41 (31.3)	27 (20.6)	26 (19.8)	26 (19.8)	3 (2.3)
Walking	292 (64.7)	1 (0.2)	8 (1.8)	36 (8.0)	109 (24.2)	5 (1.1)	6 (4.8)	47 (37.3)	22 (17.5)	22 (17.5)	24 (19.0)	5^a^ (4.0)
Appetite	389 (86.3)	1 (0.2)	9 (2.0)	20 (4.4)	32 (7.1)	0	6 (12.5)	14 (29.2)	7 (14.6)	6 (12.5)	15 (31.3)	0
Knowing what is wrong	337 (74.7)	0	8 (1.8)	20 (4.4)	81 (18.0)	5 (1.1)	6 (7.1)	8 (9.4)	6 (7.1)	10 (11.8)	54 (63.5)	1 (1.2)
Disease under control	216 (47.9)	1 (0.2)	5 (1.1)	39 (8.6)	188 (41.7)	2 (0.4)	3 (1.6)	39 (20.6)	36 (19.0)	32 (16.9)	72 (38.1)	7 (3.7)
Alive	243 (53.9)	0	3 (0.7)	20 (4.4)	183 (40.6)	2 (0.4)	7 (4.3)	10 (6.2)	14 (8.7)	15 (9.3)	103 (64.0)	12 (2.7)
Enjoy	304 (67.4)	0	3 (0.7)	45 (10.0)	96 (21.3)	3 (0.7)	13 (11.4)	18 (15.8)	25 (21.9)	24 (21.1)	30 (26.3)	4 (3.5)
Groceries	386 (85.6)	2 (0.4)	7 (1.6)	20 (4.4)	36 (8.0)	0	14 (26.9)	13 (25.0)	4 (7.7)	6 (11.5)	14 (26.9)	1 (1.9)
Wash and dress	384 (85.1)	0	3 (0.7)	19 (4.2)	43 (9.5)	2 (0.4)	18 (34.0)	10 (18.9)	4 (7.5)	3 (5.7)	16 (20.2)	2 (3.8)
Garden	365 (80.9)	4 (0.9)	14 (3.1)	21 (4.7)	46 (10.2)	1 (0.2)	10 (14.5)	18 (26.1)	16 (23.2)	11 (15.9)	14 (20.3)	0
Sports	359 (79.6)	4 (0.9)	9 (2.0)	30 (6.7)	48 (10.6)	1 (0.2)	15 (19.7)	26 (34.2)	8 (10.5)	10 (13.2)	14 (18.4)	3 (3.9)
Hobbies	374 (82.9)	1 (0.2)	8 (1.8)	22 (4.9)	46 (10.2)	0	8 (12.9)	18 (29.0)	9 (14.5)	10 (16.1)	15 (24.2)	2 (3.2)
Driving	388 (86.0)	1 (0.2)	3 (0.7)	12 (2.7)	46 (10.2)	1 (0.2)	13 (25.0)	15 (28.8)	2 (3.8)	2 (3.8)	18 (34.6)	2 (3.8)
Outings	369 (81.8)	2 (0.4)	7 (1.6)	28 (6.2)	44 (9.8)	1 (0.2)	9 (14.5)	23 (37.1)	11 (17.7)	9 (14.5)	8 (12.9)	2 (3.2)
Visiting	391 (86.7)	0	5 (1.1)	20 (4.4)	34 (7.5)	1 (0.2)	11 (24.4)	18 (40.0)	5 (11.1)	3 (6.7)	8 (17.8)	0
Home	423 (93.8)	1 (0.2)	0	7 (1.6)	20 (4.4)	0	3 (18.8)	1 (6.3)	1 (6.3)	0	10 (62.5)	1 (6.3)
Independence	377 (83.6)	1 (0.2)	1 (0.2)	18 (4.0)	52 (11.5)	2 (0.4)	11 (22.4)	13 (26.5)	7 (14.3)	10 (20.4)	7 (14.3)	1 (2.0)
Extra	432 (95.8)	0	0	2 (0.4)	17 (3.8)	0	0	5 (35.7)	2 (14.3)	2 (14.3)	3 (21.4)	2 (14.3)

^a^ Due to a random temporary error in the computer system, the items defecation (*n* = 2) and walking (*n* = 4) were not asked

At follow-up, participants sometimes mentioned that the goal was not applicable for them. This ranged from 1.6 to 34.0% per goal, except for the extra goal. Missing values are in two cases due to the participant stopping answering questions halfway through the P-BAS HOP. The item ‘alive’ had the highest number of missing values, mostly (eight times) because the participant did not know the answer. The item ‘disease under control’ had the second highest number of missing values. Regarding this question, some participants mentioned they did not know how their situation was at that moment, because they were still under treatment or waiting test results.

The PBI ranged from 0 to 3 points, with a mean of 1.71 and a standard deviation of 0.93.

### Reliability

#### Baseline questionnaire

For the test-retest reliability, 60 participants were approached. Seven times the participant refused the retest, resulting in 53 participants performing a baseline test-retest reliability. Median time between test and retest was 1 day. In 33 cases the retest was performed by another interviewer and in 20 cases with the same interviewer. We therefore decided also to distinguish between intra- and inter-rater reliability.

Of the 21 specified goals, from which participants could select, the number of discrepancies between test and retest per participant ranged from zero to a maximum of 11 (52% of the number of goals) with a median of four goals (19%). From the cases with the same interviewer, the number of discrepancies between test and retest per participant ranged from zero to seven (33%) with a median of three goals (14%). The cases with different interviewers had one (5%) to 11 (52%) discrepancies between test and retest per participant with a median of five goals (24%). Of the total of 228 discrepancies, in 100 (44%) cases the goal was selected only during the test and in 128 (56%) cases only during the retest. These proportions were the same for the intra- and inter-rater reliability.

The complete crosstabulations of all items are included in Additional File 3. Table 6 shows the weighted kappa per item in descending order. The weighted kappa for the item ‘home’ could not be calculated because of too many empty cells. Two items had substantial agreement, eight moderate agreement, seven fair agreement and three slight agreement.

**Table 6:** Cohen’s weighted kappa with quadratic weighting for baseline items in descending order

Item	Overall reliability (*n* = 50–53)				Intra-rater reliability (*n* = 19–20)				Inter-rater reliability (*n* = 31–33)			
	%^a^	Weighted Kappa (95% CI)	K_max_	K/ K_max_	%^a^	Weighted Kappa (95% CI)	K_max_	K/ K_max_	%^a^	Weighted Kappa (95% CI)	K_max_	K/ K_max_
Bowel movement	88.68	0.66 (0.37;0.96)	0.95	0.70	95.00	0.85 (0.51;1)	0.85	1	84.85	0.58 (0.21;0.95)	0.86	0.68
Walking	64.00	0.63 (0.46;0.81)	0.95	0.61	78.95	0.84 (0.72;0.97)	0.87	0.97	54.84	0.49 (0.23;0.75)	0.84	0.58
Gardening	84.91	0.55 (0.26;0.84)	0.68	0.81	85.00	0.74 (0.41;1)	nc	nc	84.85	0.39 (0.05;0.74)	0.66	0.60
Shortness of breath	60.38	0.54 (0.33;0.75)	0.97	0.56	60.00	0.56 (0.24;0.88)	0.97	0.58	60.61	0.53 (0.26;0.80)	0.91	0.58
Independence	80.77	0.54 (0.28;0.79)	0.96	0.56	85.00	0.74 (0.48;0.99)	nc	nc	78.13	0.44 (0.11;0.77)	0.86	0.51
Pain	66.04	0.52 (0.32;0.72)	0.95	0.55	75.00	0.56 (0.24;0.88)	0.83	0.67	60.61	0.49 (0.24;0.74)	0.76	0.64
Sports	75.47	0.51 (0.25;0.76)	0.75	0.68	80.00	0.65 (0.30;0.99)	0.96	0.67	72.73	0.38 (0.10;0.66)	nc	nc
Knowing what is wrong	67.92	0.48 (0.26;0.70)	0.95	0.50	60.00	0.30 (0;0.68)	0.97	0.31	72.73	0.58 (0.33;0.83)	0.88	0.66
Energy	54.72	0.43 (0.21;0.66)	0.94	0.46	65.00	0.62 (0.30;0.86)	0.62	1	48.48	0.35 (0.09;0.61)	0.64	0.55
Controlling disease	59.62	0.42 (0.21;0.63)	0.78	0.54	60.00	0.50 (0.20;0.80)	0.62	0.80	59.38	0.38 (0.11;0.66)	0.95	0.40
Groceries	77.36	0.40 (0.14;0.66)	0.95	0.42	90.00	0.85 (nc)	0.85	1	69.70	0.28 (0;0.57)	0.94	0.30
Hobbies	76.92	0.30 (0.03;0.57)	0.95	0.31	70.00	0.34 (0;0.74)	0.94	0.36	81.25	0.27 (0;0.62)	0.70	0.38
Visiting	75.47	0.29 (0.05;0.53)	0.96	0.30	75.00	0.47 (0.09;0.85)	0.86	0.55	75.76	0.14 (0;0.28)	0.95	0.14
Outings	67.31	0.28 (0.09;0.48)	0.74	0.38	70.00	0.44 (0.13;0.74)	0.44	1	65.63	0.21 (0;0.43)	0.99	0.21
Alive	62.26	0.28 (0.06;0.50)	0.91	0.31	75.00	0.45 (0.09;0.80)	0.72	0.63	54.55	0.16 (0;0.43)	0.99	0.16
Appetite	75.00	0.25 (0.07;0.43)	0.66	0.38	73.68	nc	nc	nc	75.76	0.46 (0;0.93)	0.72	0.65
Washing and dressing	73.08	0.25 (0.02;0.48)	0.96	0.26	70.00	0.29 (0;0.61)	0.72	0.40	75.00	0.23 (0;0.53)	0.83	0.28
Enjoying life	65.38	0.17 (0;0.38)	0.75	0.23	85.00	0.65 (0.34;0.97)	0.65	1	53.13	−0.12 (−0.39;0.14)	0.85	−0.14
Better	60.38	0.14 (0.01;0.27)	0.60	0.23	75.00	0.62 (0.23;1)	0.84	0.74	51.52	−0.16 (nc)	0.52	−0.31
Driving	83.02	0.05 (nc)	0.87	0.05	95.00	0.44 (0;1)	0.44	1	75.76	−0.08 (nc)	0.62	−0.14
Home	94.23	nc	nc	nc	95.00	nc	nc	nc	93.75	nc	nc	nc
Extra	nc	nc	nc	nc	nc	nc	nc	nc	nc	nc	nc	nc
Mean	72.04	0.38	0.86	0.44	77.03	0.58	0.77	0.75	69.00	0.30	0.81	0.35

^a^% = percentage of agreement, K = kappa K_max_ = maximum attainable weighted kappa CI = Confidence interval nc = not calculated

When the weighted kappa was calculated as a proportion of the maximum attainable kappa, the item ‘gardening’ had almost perfect agreement, three items had substantial agreement, seven items moderate agreement, eight fair agreement and the item ‘driving’ slight agreement.

Three participants who had a retest only mentioned an extra goal in the test, while three others only mentioned an extra goal in the retest. One participant mentioned a goal in the test and in the retest, but this was a different goal. Therefore, no kappa value was calculated for the extra option.

The mean of all the weighted kappa values showed fair agreement, when calculated as a proportion of the maximum attainable kappa, moderate agreement. The mean of the intra-rater kappa values showed moderate agreement, when calculated as a proportion of the maximum attainable kappa, substantial agreement. The mean of the inter-rater kappa values showed fair agreement.

From the participants with a baseline retest, 37 had a valid follow-up. The PBI of the retest ranged from 0 to 3, with a mean of 1.65. The overall ICC between the PBI of the test and retest was 0.77 (95% CI 0.60–0.87). The intra-rater ICC was 0.94 (95% CI 0.81–0.98)(*n* = 13), the inter-rater ICC was 0.68 (95% CI 0.40–0.86) (*n* = 24).

Asking the participants the reason for the discrepancies between test and retest, revealed several reasons: 1) Difference in interpretation at different moments, for example the participant had difficulties with walking due to shortness of breath, but did not have any problems with the legs. At the retest the participant did take into account the shortness of breath, at the test only the legs. 2) Priority is assessed differently at different moments, for example groceries are normally done by the partner, but it would be nice if the participant could help, or the pain is present but the participant could cope with it. 3) Progressive insight during the hospital admission: through more information, or the experience of a disappointing recovery, goals were lowered or suddenly became much more important. 4) In some cases the participant was not able to explain the reason.

#### Follow-up questionnaire

For the follow-up test-retest reliability, 90 participants were approached. In 11 cases the participant refused the retest, six times the participant could not be reached, for one case it was unknown why the retest was not performed. Finally, 72 participants performed a test-retest of the follow-up questionnaire. However, since only goals that were applicable were evaluated and the prevalence of some goals was quite rare, these goals had very small sample sizes. We therefore decided to compute weighted kappa values only when the sample size was ≥10 participants. Median time between test and retest was 9.5 days. In 43 cases the retest was performed by another interviewer and in 29 cases by the same interviewer. Sample sizes were too small to calculate kappa values for intra- and inter-rater reliability. Six values can be found in Additional file 4.

The complete crosstabulations of all the items are included in Additional File 4. Table 7 shows the weighted kappa in descending order. The item ‘enjoying life’ had almost perfect agreement. Two items had substantial agreement, six moderate agreement, two fair agreement and the item ‘knowing what is wrong’ slight agreement.

**Table 7:** Cohen’s weighted kappa with quadratic weighting for follow-up items in descending order (*n* = 1–51)

Item	n	% agreement	Weighted Kappa (95% CI)	K_max_	Weighted K/ K_max_
Enjoying life	17	88.24	0.88 (0.65;1)	0.98	0.91
Pain	14	42.86	0.72 (nc)	0.82	0.88
Sports	12	41.67	0.61 (0.39;0.83)	0.71	0.86
Controlling disease	29	55.17	0.59 (0.28;0.90)	0.87	0.67
Driving	10	60.00	0.55 (0.07;1)	0.97	0.56
Better	51	50.98	0.51 (0.20;0.81)	0.74	0.68
Alive	28	60.71	0.50 (0.18;0.82)	0.57	0.87
Shortness of breath	25	56.00	0.47 (0.07;0.88)	0.75	0.63
Energy	41	43.90	0.45 (0.16;0.74)	0.89	0.51
Gardening	13	46.15	0.40 (0;87)	0.74	0.54
Walking	25	36.00	0.24 (0;0.50)	0.83	0.28
Knowing what is wrong	10	70.00	0.17 (nc)	1	0.17
Bowel movement	5	40.00	nc	nc	nc
Appetite	9	77.77	nc	nc	nc
Groceries	5	0	nc	nc	nc
Washing and dressing	5	60.00	nc	nc	nc
Hobbies	6	50.00	nc	nc	nc
Visiting	4	25.00	nc	nc	nc
Outings	7	57.14	nc	nc	nc
Home	1	100	nc	nc	nc
Independence	7	14.28	nc	nc	nc
Extra	2	100	nc	nc	nc
Mean	15	53.43	0.51	0.83	0.63

*CI* Confidence interval, *nc* not calculated

When the weighted kappa was calculated as a proportion of the maximum attainable kappa, four items had almost perfect agreement, three substantial agreement, three moderate agreement, one fair agreement and one slight agreement.

For ten items the sample size was too small to calculate a valid kappa. The percentage of agreement for these items varied widely from zero for groceries to one hundred for home and the extra goal, although these last two items were only answered by one and two participants, respectively.

The mean of all the weighted kappa values showed a moderate agreement, when calculated as a proportion of the maximum attainable kappa, a substantial agreement.

The PBI of the retest ranged from 0 to 3 points, with a mean of 1.77. The overall ICC between the PBI of the test and retest was 0.62 (96%CI 0.45–0.74). The intra-rater ICC was 0.59 (95% CI 0.29–0.78), the inter-rater ICC was 0.64 (95% CI 0.42–0.79).

### Validity

#### Baseline questionnaire

All baseline hypotheses were confirmed. Table 2 shows the test statistics and the complete descriptive information is shown in Additional file 5.

The 50 cases selected for the open question mentioned 110 goals in total. Of these, 23 goals could not be coded as an item in the P-BAS HOP because they were too vague to categorise or the goal did not exist in the P-BAS HOP and were therefore coded as ‘other’. An example of a vague goal was: ‘that it will be the way it was’, an example of a goal that did not exist in the P-BAS HOP was: ‘that I can lift my grandson again’. We consequently analysed the agreement between the codes and the answers given in the P-BAS HOP of 87 goals and found an agreement of 75%. An overview of the number of items coded and the amount of agreement is given in Table 8.

**Table 8:** Coding of open questions and agreement with P-BAS HOP in descending order of frequency

Code	Frequency coded	Agreement n (%)	No agreement n (%)
Other	23	n.a.	n.a.
Controlling disease	16	14 (88)	2 (13)
Pain	9	6 (67)	3 (33)
Shortness of breath	8	8 (100)	0
Walking	8	7 (88)	1 (13)
Independence	8	5 (63)	3 (38)
Better	7	7 (100)	0
Sports	7	3 (43)	4 (57)
Alive	6	4 (67)	2 (33)
Energy	5	5 (100)	0
Outings	5	2 (40)	3 (60)
Hobbies	3	1 (33)	2 (67)
Garden	2	1 (50)	1 (50)
Knowing what is wrong	1	1 (100)	0
Groceries	1	1 (100)	0
Driving	1	0	1 (100)
Bowel movements	0	n.a.	n.a.
Appetite	0	n.a.	n.a.
Enjoy	0	n.a.	n.a.
Wash and dress	0	n.a.	n.a.
Visiting	0	n.a.	n.a.
Home	0	n.a.	n.a.
Total	110	65 (75)	22 (25)

#### Follow-up questionnaire

Six hypotheses did not meet the assumptions for Cramér’s V, because the number of people experiencing a deterioration on that item was very low. For four of these hypotheses the descriptive trend was in the right direction. From six of the first 12 hypotheses that were calculated, four were confirmed and two were rejected. Table 3 shows the test statistics and the complete descriptive information is shown in Additional File 6.

Of the 50 cases selected at baseline for comparing open questions, 41 had a follow-up. This resulted in 40 dyads of coded open goals and P-BAS HOP items with a follow-up. The correlation between the answers on the open question and the corresponding P-BAS HOP item was 0.71.

### Responsiveness

For the anchor question ‘How much have you benefited from the admission?’ Thirteen (4%) of the respondents did not know what to answer. Of the valid responses, 15 (5%) of the respondents answered ‘not at all’, 15 (5%) ‘a little bit’, 44 (13%) ‘somewhat’, 142 (43%) much, and 113 (34%) very much.

The Spearman’s correlation coefficient between the PBI and the anchor question was 0.51.

### Interpretability

**Fig. 2:**
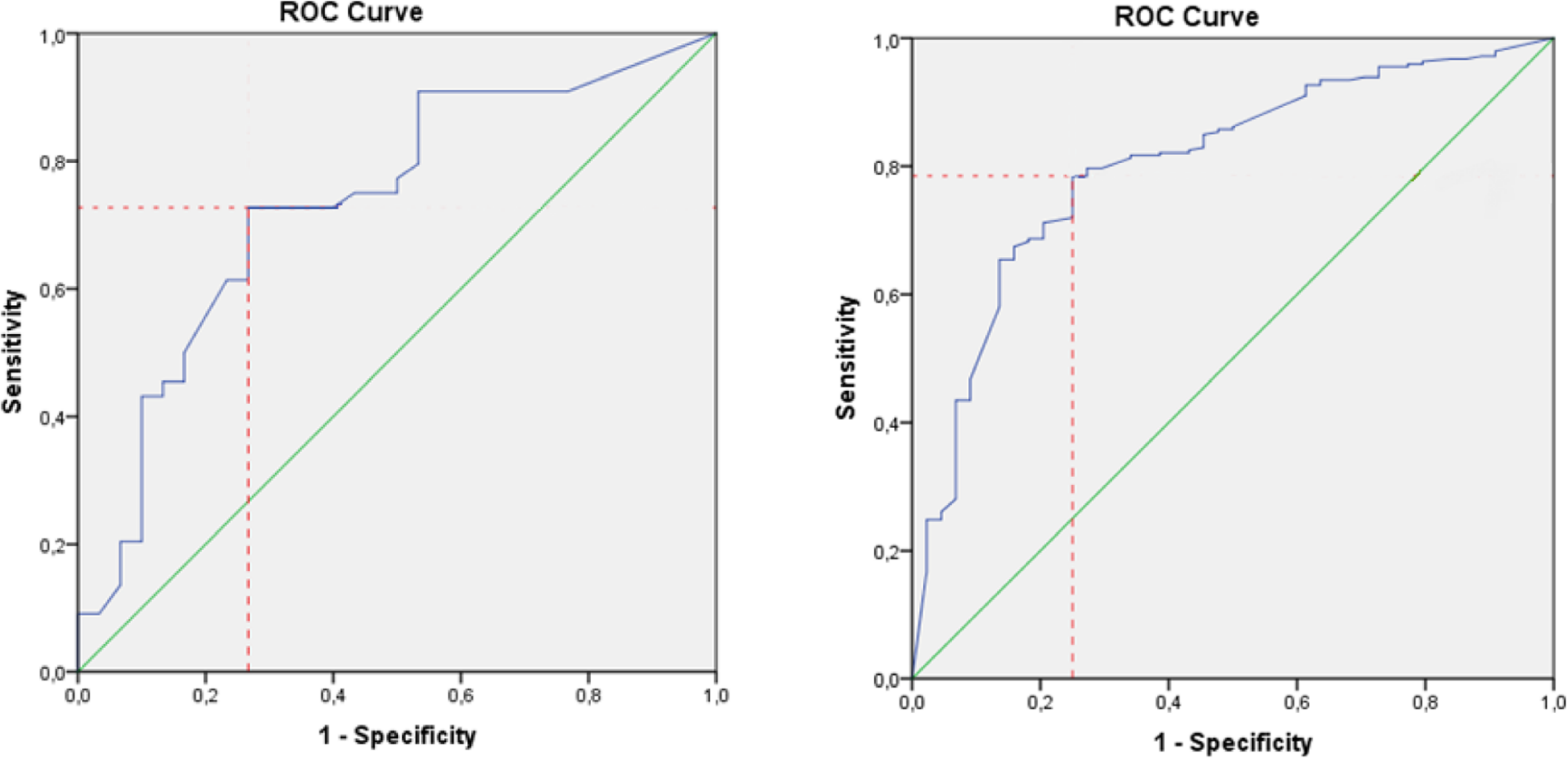
ROC curve of ‘no benefit’ (left, *n* = 74, AUC = 0.73) and ‘benefit’ (right, *n* = 290, AUC = 0.80) with optimal cut-off point. ROC = receiver operating characteristic, AUC = Area under de curve

Figure 2 shows on the left side the ROC curve of ‘no important benefit’, with an area under the curve of 0.73. The optimal cut-off point for ‘no important benefit’ was set at a sensitivity value of 73% and a specificity of 73%, resulting in an MIC of 0.7 points on the PBI.

**Fig. 3:**
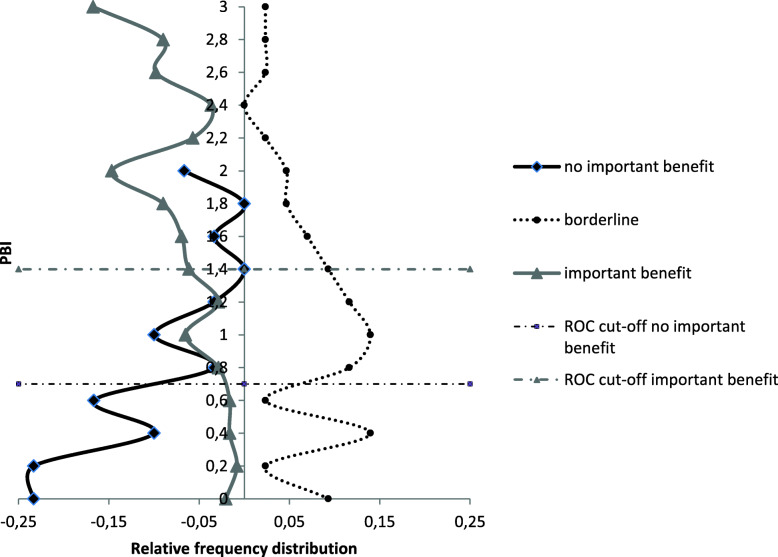
Anchor-based minimal important change (MIC) distribution. PBI = Patient Benefit Index

The right side of Fig. 2 shows the ROC curve of ‘important benefit’, with an area under the curve of 0.80. The optimal cut-off point for ‘important benefit’ was set at a sensitivity value of 79% and a specificity of 75%, resulting in a MIC of 1.4 points on the PBI. This means the PBI values between 0.7 and 1.4 are considered as ‘borderline benefit’. The anchor-based MIC distribution is displayed in Fig. 3.

## Discussion

In this study we tested the reliability, validity, responsiveness and interpretability of the Patient Benefit Assessment scale (P-BAS HOP), which was designed to identify the goals of the individual patient and to measure his/her relevant outcomes. The results are mixed.

The reliability of the individual items of the baseline questionnaire can be summarised as fair to moderate. Participants varied regularly in which goals they considered important. This could have several causes. Firstly,, although sample sizes being small, the intra-rater reliability of the baseline test appeared to be much better than inter-rater. It could have happened that the interviewer unintentionally influenced a participant when remembering the answer from the other day, but it is more probable that there is much variation between instructions given by various interviewers. This could be caused by not having all questions written out, giving more autonomy to the interviewer, or the instructions may have been insufficient. Secondly, a hospital admission is a highly unstable and unpredictable period. Symptoms vary, people receive treatments and medical information which can change their priorities. Thirdly, the definition of a problem or limitation was perhaps not very clear, since this could have been at the moment of interview, or at the moment of admission, or could have been a potential limitation. This could cause large differences in the crosstabulations: when someone, for example, declares at the test in the first step that an item does not apply, the answer is automatically doesn’t apply/not important at all, while when saying in the retest it does apply the participant goes further to the second step and can indicate there that it is ‘very important’. Fourthly, choosing which goals or items are relevant, is very different from usual questionnaires where the objective is to assess, for example, health status. When comparing the P-BAS HOP with other instruments where participants choose their own domains, it is seen that choosing other domains in the retest is common. For example in the ‘schedule for the evaluation of individual quality of life’ (SEIQoL-DW), 35 to 81% of the participants choose new domains [19, 20]. In the Patient-Generated Index (PGI) participants have to choose a maximum of five domains and the mean number of change in the retest was 1.7. 21% of the participants chose three to five new domains [20, 21].

A more technical explanation for the low kappa values, is that as a result of the individual approach of the tool, the percentage of ‘doesn’t apply to me’ is often high, resulting in very homogeneous samples, causing low kappa values [11, 12, 22].

Although the reliability of the individual items of the baseline questionnaire is fair to moderate, the ICC between the PBI of the test and retest was 0.77, which is acceptable. This means that even though not all participants are very consistent in their choice of goals, this does not lead to very deviating PBI-scores. This could be explained by the fact that many people differ only in a few goals between test and retest and that there exist moderate to strong correlations between the achievement of many goals (data not shown).

The reliability of the follow-up questionnaire is better than the baseline with a mean weighted kappa of 0.51. Participants were probably in a more stable situation during follow-up, although we have not asked whether anything had changed between test and retest. However, the variation between test and retest items on follow-up had more impact on the ICC, which was 0.62 and therefore not satisfactory. The follow-up intra- and inter-rater reliability were similar. This could be caused by having all questions written out at follow-up, leaving less room for variation between interviewers.

From the hypotheses for baseline validity, almost all hypotheses were confirmed. This suggests participants are likely to choose goals which are relevant for them. On the other hand, this is contradicted in the follow-up, where participants often stated that the goal was not applicable for them, for the goal ‘washing and dressing’ this was even 34%. This could have several causes: first, the P-BAS HOP does not discriminate between preservation and improvement, so the goal could have been to preserve a function, but this is not clear in the questioning, especially through use of the word ‘again’. Second, participants may have forgotten in what poor condition they were during admission, therefore ignoring how much they have improved. In the literature, this is called response shift or recall bias, and occurs more frequently opposite, so patients underestimate afterwards their condition during admission [23–25]. However, Hinz et al. showed that around 20 to 30% of the patients afterwards overestimated their condition during admission [24]. A third explanation could be that it is unclear which time period the participants had to compare with: during hospitalisation, for example, participant were unable to wash and dress themselves, but before admission this was not a problem. Compared to the situation at admission it was an improvement, but compared to the situation before, the hospitalisation did not make a difference.

The agreement between goals coded in the open questions and the P-BAS HOP items was 75%, which we considered just valid. This could partly be due to ambiguity: some goals were difficult to code. For example: the goal ‘that I can be part of club life’ we coded as ‘hobbies’, but we were not sure what kind of club this participant wanted to be part of and whether this could be seen as a hobby or not. Nevertheless, there were also examples of situations where there was clear disagreement between the goal set by the participant in the open question and the P-BAS HOP. For example, a person stated in the open question ‘being able to work in the garden’ and in the P-BAS HOP the item ‘gardening’ was marked as ‘not applicable’. This could be caused by the first part of the baseline questionnaire where the participant states whether experiencing or expecting limitations regarding that subject. Apparently a subject does not need to be an actual problem or limitations to be a goal.

A limitation of the method of comparing goals in the open question and the P-BAS HOP, is that participants could mention several goals, but we treated the coded goals and the answers in the P-BAS HOP as if they were independent.

For the testing of the validity in the follow-up, we were limited by small sample sizes and the fact that only small numbers of people deteriorated on the Katz-15, EQ-5D or MSPP between baseline and follow-up. Other studies reported higher amounts of deterioration from around one third of the older patients [26–28]. We probably had a selection bias of the most fit patients wanting to participate.

Of the follow-up hypotheses that were tested, one third were rejected, we therefore have to conclude that the validity of the follow-up questions was weak. This could be a result of recall bias, but also because participants did not know which time period they had to compare with. We did not observe difficulties with validity of the follow-up questionnaire in the Three Step Test Interviews (TSTI) during the pilot [4], but this could be due to the fact we did the TSTI at discharge and not when people were back home for several weeks.

Although the validity of the follow-up questionnaire was weak, the PBI could be considered valid, so the sum of the achievement of all goals weighted for their importance gives a good representation of the benefit the participant experienced by the hospital admission. A disadvantage of an anchor-based method is that the conclusion is always dependent on the anchor chosen [17]. Many participants gave an explanation to their answer to the anchor question, and this revealed that the conclusion of how much benefit the participant had, was not always based on the goals achieved, but could also be based on other indicators, for example how kind the hospital staff was.

For the interpretability we constructed cut-off values for relevant benefit, but one should take into account that a cut-off is in reality not an absolute value and could be dependent on the sample [18].

### Limitations

The sample size of the reliability studies was quite low, especially when taking into account the homogenous samples at baseline. Therefore, the confidence intervals around the kappa values were often large. Another result of the homogenous samples at baseline, is that the numbers of the middle categories are quite low, not meeting the criterion of a minimum of 10 cases in the margins [29]. We therefore also computed kappa values for 2 × 2 tables, by combining the categories ‘doesn’t apply/not at all important’ with ‘somewhat important’ and ‘quite important’ with ‘very important’. This showed similar results, although still not all margins had 10 cases (data not shown). At follow-up the problem of the low sample sizes was larger, since only goals that applied were evaluated and some goals were only chosen by a few participants.

Since the P-BAS HOP was administered on paper, interviewers had to manually circle the goals to ask in the second part, based on the subjects indicated as applicable in the first part. This lead sometimes to the omission of a goal by forgetting to circle a goal.

The time between discharge and follow-up was 3 months, which is quite long if patients have to indicate to what extent the hospitalisation helped to achieve the set goals. In the meantime there could be various other factors which have influenced the result and which are difficult to disentangle from the hospital admission.

## Conclusions and recommendations

Although the concept seems promising, the reliability and validity of the P-BAS HOP appeared to be not yet satisfactory in this format. We therefore recommend adapting the P-BAS HOP, subsequently re-evaluating the reliability and validity, as follows: modify the first step in which the participant is asked whether experiencing a problem or limitation with a subject, discriminate between prevention, preservation and improvement, and remove the word ‘again’. Also reformulate the questions in the follow-up questionnaire or make clear to which time frame they refer. A good instruction and supervision of the interviewers appeared to be very important to reduce variability between interviewers. Finally, a computer assisted system could reduce missing values.

## Supplementary Information

**Additional file 1.**

**Additional file 2.**

**Additional file 3.**

**Additional file 4.**

**Additional file 5.**

**Additional file 6.**

## Data Availability

The datasets used and/or analysed during the current study are available from the corresponding author on reasonable request.

## Abbreviations

ADL: Activities of Daily Living
ICC: Intraclass Correlation Coefficient
MIC: Minimal Important Change
MSPP: Maastricht Social Participation Profile
NRS: Numeric Rating Scale
P-BAS HOP: Patient Benefit Assessment Scale for Hospitalised Older Patients
PBI: Patient Benefit Index
ROC: Receiver Operating Characteristic
RSCL: Rotterdam Symptom Checklist
SF-36: 36-Item Short Form Survey Instrument
TSTI: Three Step Test Interviews
VAS: Visual Analogue Scale
VMS: Safety Management Programme
