# Supplementary material for: Reliability and validity of the Patient Benefit Assessment Scale for Hospitalised Older Patients (P-BAS HOP)
Source: BMC Geriatr. 2021 Mar 1;21:149. doi: 10.1186/s12877-021-02079-z (PMC7923656; doi:10.1186/s12877-021-02079-z)
Supplement: Supplementary file 1 — Additional file 1. [file 12877_2021_2079_MOESM1_ESM.docx]

**Additional file 1. Questionnaires used to test the construct validity**

**Reliability and validity of the Patient Benefit Assessment Scale for Hospitalised Older Patients (P-BAS HOP)**

**Authors:**

1. Maria Johanna van der Kluit, MSc RN (Corresponding author)

University of Groningen, University Medical Center Groningen, University Center for Geriatric Medicine, Hanzeplein 1, 9700 RB Groningen, The Netherlands

m.j.van.der.kluit@umcg.nl

+31503613921

1. Geke J. Dijkstra, PhD

University of Groningen, University Medical Center Groningen, Department of Health Sciences, Applied Health Research, Groningen, The Netherlands

NHL Stenden University of Applied Sciences, Research Group Living, Wellbeing and Care for Older People, Leeuwarden, The Netherlands

[g.j.dijkstra@umcg.nl](mailto:g.j.dijkstra@umcg.nl)

1. Sophia E. de Rooij, MD PhD

University of Groningen, University Medical Center Groningen, University Center for Geriatric Medicine, Groningen, The Netherlands

Medical Spectrum Twente, Medical School Twente, Enschede, The Netherlands

sejaderooij@gmail.com

**Additional file 1. Questionnaires used to test the construct validity**

Details of the constructs and questionnaires summarised in Table 1.

**Dutch VMS screening program (VMS)**

The VMS questionnaire, which is developed as part of the Dutch National Hospital Safety Management Programme, consists of four instruments: Activities of daily living (ADL), falls, undernutrition and delirium (1). For the hypotheses to test the validity only the question about appetite is analysed: The participants were asked whether they experienced a decrease of appetite during the last month (yes/no). The questions were asked at baseline and follow-up.

**Rotterdam Symptom Checklist (RSCL)**

The RSCL was developed to measure symptoms reported by cancer patients participating in clinical research. It consists of a broad list with symptoms concerning psychological and physical distress (2). Originally, the symptoms are on a four points Likert scale, but we dichotomised the symptoms into present or absent on admission day.

**Pain and Fatigue Numeric Rating Scale (NRS)**

Participants were asked to rate their pain and fatigue as experienced at the moment of interview. The scale runs from 0: no pain/ fatigue at all to 10: the worst imaginable pain/ fatigue.

**EQ-5D**

The EQ-5D is a standardised, non-disease-specific instrument for describing and valuing health-related quality of life. It consists of five dimensions and a Visual Analogue Scale (VAS). The dimensions are mobility, self-care, usual activities, pain/discomfort and anxiety/depression, with three answer options each: no problems, some problems and extreme problems. The VAS, often referred to as the EuroQol ‘thermometer’, has an endpoint of 100 for best imaginable health state and 0 for worst imaginable health state (3). Participants were asked, during the baseline interview, to indicate their health state two weeks prior to hospital admission and, during the follow-up interview, to indicate their health state at the day of interview.

**Admission reason**

Admission reason was obtained from the medical record, recorded by the attending physician and retrieved by a medical student. Options were acute/elective; diagnostic/curative/palliative. When a participant gave no informed consent for record insight, this was labelled as ‘unknown’.

**Katz-15 scale**

The Katz-15 scale consists of fifteen items regarding basic activities of daily living such as the need for help with bathing and Instrumental Activities of Daily Living such as shopping. The answer options are dichotomous (4). Participants were asked, during the baseline interview, to indicate their functioning two weeks prior to hospital admission and, during the follow-up interview, to indicate their functioning at the day of interview.

**Maastricht Social Participation Profile (MSPP)**

The first half (46%) of the sample answered the MSPP (5). The MSPP is an instrument measuring the actual social participation by older adults. Participation is operationalised in part A: consumptive participation and formal social participation, and part B and C: informal social participation. In the original instrument informal social participation was split into participation with friends or acquaintances (part B) and participation with family (part C). In our study, we combined the items of B and C, resulting in a total of 18 items. For each item it was asked how frequently it was performed during the past four weeks with answer options 0, 1-3, 4-8, 9+ (5). We used individual items of the MSPP for the validation, but computed one sum score: All items concerning a day trip, which are items 3-8 of part A and items 4 and 5 of part B/C, are summed into ‘MSPP-daytrip’.

**36-Item Short Form Survey Instrument (SF-36) – Social functioning**

The second half of the participants (50%) answered the question ‘During the past four weeks, how much of the time has your physical health or emotional problems interfered with your social activities (like visiting with friends, relatives, etc.)?’, which is part of the SF-36 Health Survey, but is used as a single item in our survey. The answer options were: none of the time, a little of the time, some of the time, most of the time, all of the time (6).

**Goals on hospital admission**

Open question to the participant at baseline: ‘What do you hope to accomplish with this hospitalisation?’ The goal stated by the participant was repeated at follow-up and asked to what extent the participant had accomplished the goal with the answer options: ‘not at all’, ‘somewhat’, ‘moderately’ ‘quite’, or ‘completely’.
