# Supplementary material for: Reliability and validity of the Patient Benefit Assessment Scale for Hospitalised Older Patients (P-BAS HOP)
Source: BMC Geriatr. 2021 Mar 1;21:149. doi: 10.1186/s12877-021-02079-z (PMC7923656; doi:10.1186/s12877-021-02079-z)
Supplement: Supplementary file 2 — Additional file 2. [file 12877_2021_2079_MOESM2_ESM.docx]

**Additional file 2. Other questionnaires. Baseline, Follow-up and Change scores**

**Reliability and validity of the Patient Benefit Assessment Scale for Hospitalised Older Patients (P-BAS HOP)**

**Authors:**

1. Maria Johanna van der Kluit, MSc RN (Corresponding author)

University of Groningen, University Medical Center Groningen, University Center for Geriatric Medicine, Hanzeplein 1, 9700 RB Groningen, The Netherlands

[m.j.van.der.kluit@umcg.nl](mailto:m.j.van.der.kluit@umcg.nl)

+31503613921

1. Geke J. Dijkstra, PhD

University of Groningen, University Medical Center Groningen, Department of Health Sciences, Applied Health Research, Groningen, The Netherlands

NHL Stenden University of Applied Sciences, Research Group Living, Wellbeing and Care for Older People, Leeuwarden, The Netherlands

[g.j.dijkstra@umcg.nl](mailto:g.j.dijkstra@umcg.nl)

1. Sophia E. de Rooij, MD PhD

University of Groningen, University Medical Center Groningen, University Center for Geriatric Medicine, Groningen, The Netherlands

Medical Spectrum Twente, Medical School Twente, Enschede, The Netherlands

sejaderooij@gmail.com

**Additional file 2. Other questionnaires. Baseline, Follow-up and Change scores**

|  | Baseline  n=451 | Follow-up  n=344 | Change (FU –B) |
| --- | --- | --- | --- |
| Item | n (%) | n (%) | Deterioration (d) : n (%)  No change (n): n (%)  Improvement (i): n (%) |
| VMS - Lack of appetite  missing | 97 (22)  4 | 67 (20)  0 | n.a. |
| RSCL - Lack of appetite  missing | 114 (26)  9 | n.a. | n.a. |
| RSCL - Tiredness  missing | 234 (52)  8 | n.a. | n.a. |
| RSCL - Lack of energy  missing | 139 (31)  10 | n.a. | n.a. |
| RSCL – Constipation  missing | 39 (9)  8 | n.a. | n.a. |
| RSCL – Diarrhoea  missing | 32 (7)  9 | n.a. | n.a. |
| RSCL – Shortness of breath  missing | 194 (44)  9 | n.a. | n.a. |
| Katz-15 – Bathing  missing | 54 (12)  0 | 44 (13)  0 | d: 17 (5)  n: 316 (92)  i: 11 (3) |
| Katz-15 – Dressing  missing | 41 (9)  0 | 25 (7)  0 | d: 8 (2)  n: 322 (94)  i: 14 (4) |
| Katz-15 – Walking  missing | 122 (27)  0 | 101 (29)  0 | d: 23 (7)  n: 309 (90)  i: 12 (4) |
| Katz-15 – Travelling  missing | 43 (10)  1 | 61 (18)  1 | d: 44 (13)  n: 287 (84)  i: 12 (4) |
| Katz-15 – Shopping  missing | 71 (16)  0 | 70 (20)  1 | d: 41 (12)  n: 286 (83)  i: 16 (5) |
| EQ-5D – Mobility  No problems  Some problems  Confined to bed  missing | 192 (43)  236 (52)  16 (4)  7 | 158 (46)  1179 (52)  5 (2)  2 | d: 51 (15)  n: 221 (65)  i: 67 (20) |
| EQ-5D – Self-care  No problems  Some problems  Unable  missing | 373 (83)  61 (14)  13 (3)  4 | 292 (85)  38 (11)  14 (4)  0 | d: 32 (9)  n: 282 (82)  i: 29 (9) |
| EQ-5D – Pain/discomfort  No  Moderate  Extreme  missing | 225 (50)  164 (36)  58 (13)  4 | 159 (46)  161 (47)  23 (7)  1 | d: 75 (22)  n: 193 (56)  i: 74 (22) |
| EQ-5D – VAS  missing | Mean: 65.66  SD: 20.43  Range: 0 - 100  10 | Mean: 67.02  SD: 21.07  Range: 0 - 100  8 | Mean: 1.64  SD: 21.64  Range: -82 - 85 |
| MSPP – Sports  0  1-3  4-8  9+  missing | 174 (85)  3 (2)  22 (11)  5 (3)  5 | 122 (79)  3 (2)  20 (13)  9 (6)  3 | d: 9 (6)  n: 122 (82)  i: 17 (12) |
| MSPP – Cultural event  0  1-3  4-8  9+  missing | 160 (78)  34 (17)  10 (5)  0  5 | 102 (67)  42 (28)  8 (5)  1 (1)  4 | d: 9 (6)  n: 105 (71)  i: 33 (23) |
| MSPP – Eaten out  0  1-3  4-8  9+  missing | 81 (40)  88 (43)  28 (14)  7 (3)  5 | 55 (36)  78 (51)  17 (11)  3 (2)  4 | d: 32 (22)  n: 87 (59)  i: 28 (19) |
| MSPP – Pub  0  1-3  4-8  9+  missing | 121 (60)  49 (24)  28 (14)  5 (3)  6 | 87 (57)  43 (28)  19 (12)  4 (3)  4 | d: 38 (26)  n: 67 (46)  i: 41 (28) |
| MSPP – Public event  0  1-3  4-8  9+  missing | 163 (81)  33 (16)  6 (3)  0  7 | 116 (76)  33 (22)  3 (2)  0  5 | d: 18 (13)  n: 101 (70)  i: 25 (17) |
| MSPP – Games  0  1-3  4-8  9+  missing | 182 (89)  12 (6)  8 (4)  2 (1)  5 | 119 (78)  18 (12)  14 (9)  2 (1)  4 | d: 10 (7)  n: 115 (78)  i: 22 (15) |
| MSPP – Day trip  0  1-3  4-8  9+  missing | 196 (44)  8 (4)  0  0  5 | 143 (94)  10 (7)  0  0  4 | d: 5 (3)  n: 136 (91)  i: 8 (5) |
| MSPP – Visiting  0  1-3  4-8  9+  missing | 48 (11)  53 (26)  70 (34)  33 (16)  5 | 35 (23)  35 (23)  55 (36)  28 (18)  4 | d: 36 (25)  n: 70 (48)  i: 41 (28) |
| MSPP – Outing considerable physical effort  0  1-3  4-8  9+  missing | 169 (83)  19 (9)  13 (6)  3 (2)  5 | 119 (78)  19 (12)  15 (10)  0  4 | d: 21 (14)  n: 104 (23)  i: 24 (16) |
| MSPP – Outing little physical effort  0  1-3  4-8  9+  missing | 124 (61)  50 (25)  22 (11)  8 (4)  5 | 83 (54)  38 (25)  25 (16)  7 (5)  4 | d: 33 (22)  n: 70 (48)  i: 44 (30) |
| MSPP-Daytrip (sum score)  missing | Median: 2  IQR: 1-4  Range: 0-11  5 | Median: 3  IQR: 1-5  Range: 0-14  4 | Mean: 0.36  SD: 2.94  Range: -8 -10 |
| Fatigue – NRS  missing | Median: 4  IQR: 2-6  Range: 0-10  7 | Median: 3  IQR: 1-5  Range: 0-10  1 | Mean: -0.91  SD: 3.03  Range: -10 - 7 |
| Pain – NRS  missing | Median: 0  IQR: 0-3  Range: 0-10  9 | n.a. | n.a. |
| SF-36 - Social activities  missing | Median: 0  IQR: 0-2  Range: 0-4  3 | Median: 0  IQR: 0-2  Range: 0-4  4 | Mean: 0.03  SD: 1.64  Range: -4 - 4 |
| Living situation  Independent  Sheltered accommodation  Senior home  Nursing home  missing | 432 (96)  14 (3)  3 (1)  2 (0)  0 | 329 (96)  8 (2)  2 (1)  3 (1)  0 | No change: 331 (96)  To more dependent: 8 (2)  To more independent: 5 (1) |

EQ-5D – VAS: Higher number is better perceived health. Change: positive is increase in perceived health

Fatigue –NRS: numeric rating scale. Higher number is more fatigue. Change: positive is increase in experienced fatigue

Pain–NRS: numeric rating scale. Higher number is more experienced pain.

SF-36: higher number is more experienced limitation. Change: positive is increase in interference with social activities.

SD = Standard deviation, IQR = Interquartile range
