# Supplementary material for: Reliability and validity of the Patient Benefit Assessment Scale for Hospitalised Older Patients (P-BAS HOP)
Source: BMC Geriatr. 2021 Mar 1;21:149. doi: 10.1186/s12877-021-02079-z (PMC7923656; doi:10.1186/s12877-021-02079-z)
Supplement: Supplementary file 3 — Additional file 3. [file 12877_2021_2079_MOESM3_ESM.docx]

**Additional file 3. Crosstabulations of test-retest Baseline**

**Reliability and validity of the Patient Benefit Assessment Scale for Hospitalised Older Patients (P-BAS HOP)**

**Authors:**

1. Maria Johanna van der Kluit, MSc RN (Corresponding author)

University of Groningen, University Medical Center Groningen, University Center for Geriatric Medicine, Hanzeplein 1, 9700 RB Groningen, The Netherlands

[m.j.van.der.kluit@umcg.nl](mailto:m.j.van.der.kluit@umcg.nl)

+31503613921

1. Geke J. Dijkstra, PhD

University of Groningen, University Medical Center Groningen, Department of Health Sciences, Applied Health Research, Groningen, The Netherlands

NHL Stenden University of Applied Sciences, Research Group Living, Wellbeing and Care for Older People, Leeuwarden, The Netherlands

[g.j.dijkstra@umcg.nl](mailto:g.j.dijkstra@umcg.nl)

1. Sophia E. de Rooij, MD PhD

University of Groningen, University Medical Center Groningen, University Center for Geriatric Medicine, Groningen, The Netherlands

Medical Spectrum Twente, Medical School Twente, Enschede, The Netherlands

sejaderooij@gmail.com

**Additional file 3. Crosstabulationss of test-retest Baseline. Table 1 Overall test-retest**

| Item | Test  Retest | Doesn’t apply/ not at all important | Somewhat important | Quite important | Very important | Total | Weighted Kappa (95% CI) |
| --- | --- | --- | --- | --- | --- | --- | --- |
| Better | Doesn’t apply/ not at all important | **3** | 0 | 0 | 10 | 13 | 0.14 (0.01;0.27) |
|  | Somewhat important | 0 | **0** | 0 | 0 | 0 |  |
|  | Quite important | 1 | 1 | **6** | 3 | 11 |  |
|  | Very important | 2 | 0 | 4 | **23** | 29 |  |
|  | Total | 6 | 1 | 10 | 36 | **53** |  |
|  | | | | | | | |
| Energy | Doesn’t apply/ not at all important | **15** | 2 | 4 | 4 | 25 | 0.43 (0.21;0.66) |
|  | Somewhat important | 1 | **0** | 0 | 0 | 1 |  |
|  | Quite important | 2 | 1 | **7** | 3 | 13 |  |
|  | Very important | 3 | 0 | 4 | **7** | 14 |  |
|  | Total | 21 | 3 | 15 | 14 | **53** |  |
|  | | | | | | | |
| Pain | Doesn’t apply/ not at all important | **26** | 0 | 4 | 4 | 34 | 0.52 (0.32;0.72) |
|  | Somewhat important | 0 | **1** | 0 | 0 | 1 |  |
|  | Quite important | 2 | 0 | **0** | 2 | 4 |  |
|  | Very important | 2 | 2 | 2 | **8** | 14 |  |
|  | Total | 30 | 3 | 6 | 14 | **53** |  |
|  | | | | | | | |
| Bowel movement | Doesn’t apply/ not at all important | **41** | 1 | 2 | 1 | 45 | 0.66 (0.37;0.96) |
|  | Somewhat important | 0 | **0** | 0 | 0 | 0 |  |
|  | Quite important | 1 | 0 | **3** | 0 | 4 |  |
|  | Very important | 1 | 0 | 0 | **3** | 4 |  |
|  | Total | 43 | 1 | 5 | 4 | **53** |  |
|  | | | | | | | |
| Shortness of breath | Doesn’t apply/ not at all important | **19** | 0 | 2 | 3 | 24 | 0.54 (0.33;0.75) |
|  | Somewhat important | 2 | **1** | 1 | 1 | 5 |  |
|  | Quite important | 1 | 0 | **4** | 4 | 9 |  |
|  | Very important | 3 | 1 | 3 | **8** | 15 |  |
|  | Total | 25 | 2 | 10 | 16 | **53** |  |
| Item | Test  Retest | Doesn’t apply/ not at all important | Somewhat important | Quite important | Very important | Total | Weighted Kappa (95% CI) |
| Walking | Doesn’t apply/ not at all important | **19** | 1 | 3 | 3 | 22 | 0.63 (0.46;0.81) |
|  | Somewhat important | 1 | **0** | 0 | 0 | 1 |  |
|  | Quite important | 1 | 1 | **1** | 2 | 5 |  |
|  | Very important | 2 | 0 | 4 | **12** | 18 |  |
|  | Total | 23 | 2 | 8 | 17 | **50** |  |
|  | | | | | | | |
| Appetite | Doesn’t apply/ not at all important | **38** | 2 | 6 | 0 | 46 | 0.25 (0.07;0.43) |
|  | Somewhat important | 2 | **0** | 0 | 0 | 2 |  |
|  | Quite important | 2 | 0 | **1** | 0 | 3 |  |
|  | Very important | 0 | 0 | 1 | **0** | 1 |  |
|  | Total | 42 | 2 | 8 | 0 | **52** |  |
|  | | | | | | | |
| Knowing what is wrong | Doesn’t apply/ not at all important | **28** | 2 | 2 | 3 | 35 | 0.48 (0.26;0.70) |
|  | Somewhat important | 0 | **0** | 0 | 0 | 0 |  |
|  | Quite important | 2 | 0 | **1** | 1 | 4 |  |
|  | Very important | 5 | 0 | 2 | **7** | 14 |  |
|  | Total | 35 | 2 | 5 | 11 | **53** |  |
|  | | | | | | | |
| Controlling disease | Doesn’t apply/ not at all important | **15** | 0 | 2 | 8 | 25 | 0.42 (0.21;0.63) |
|  | Somewhat important | 1 | **0** | 1 | 0 | 2 |  |
|  | Quite important | 1 | 1 | **2** | 2 | 6 |  |
|  | Very important | 3 | 0 | 2 | **14** | 19 |  |
|  | Total | 20 | 1 | 7 | 24 | **52** |  |
|  | | | | | | | |
| Alive | Doesn’t apply/ not at all important | **22** | 0 | 1 | 9 | 32 | 0.28 (0.06;0.50) |
|  | Somewhat important | 0 | **0** | 0 | 0 | 0 |  |
|  | Quite important | 0 | 1 | **2** | 1 | 4 |  |
|  | Very important | 7 | 0 | 1 | **9** | 17 |  |
|  | Total | 29 | 1 | 4 | 19 | **53** |  |

| Item | Test  Retest | Doesn’t apply/ not at all important | Somewhat important | Quite important | Very important | Total | Weighted Kappa (95% CI) |
| --- | --- | --- | --- | --- | --- | --- | --- |
| Enjoying life | Doesn’t apply/ not at all important | **31** | 0 | 2 | 8 | 41 | 0.17 (0;0.38) |
|  | Somewhat important | 0 | **0** | 0 | 0 | 0 |  |
|  | Quite important | 1 | 1 | **1** | 1 | 4 |  |
|  | Very important | 4 | 0 | 1 | **2** | 7 |  |
|  | Total | 36 | 1 | 4 | 11 | **52** |  |
|  | | | | | | | |
| Groceries | Doesn’t apply/ not at all important | **38** | 1 | 1 | 3 | 43 | 0.40 (0.14;0.66) |
|  | Somewhat important | 1 | **1** | 0 | 0 | 2 |  |
|  | Quite important | 0 | 0 | **1** | 1 | 2 |  |
|  | Very important | 3 | 0 | 2 | **1** | 6 |  |
|  | Total | 42 | 2 | 4 | 5 | **53** |  |
|  | | | | | | | |
| Washing and dressing | Doesn’t apply/ not at all important | **36** | 1 | 0 | 5 | 42 | 0.25 (0.02;0.48) |
|  | Somewhat important | 0 | **0** | 0 | 0 | 0 |  |
|  | Quite important | 2 | 0 | **1** | 0 | 3 |  |
|  | Very important | 3 | 1 | 2 | **1** | 7 |  |
|  | Total | 41 | 2 | 3 | 6 | **52** |  |
|  | | | | | | | |
| Gardening | Doesn’t apply/ not at all important | **39** | 0 | 0 | 1 | 40 | 0.55 (0.26;0.84) |
|  | Somewhat important | 0 | **2** | 0 | 0 | 2 |  |
|  | Quite important | 1 | 1 | **3** | 0 | 5 |  |
|  | Very important | 3 | 0 | 2 | **1** | 6 |  |
|  | Total | 43 | 3 | 5 | 2 | **53** |  |
|  | | | | | | | |
| Sports | Doesn’t apply/ not at all important | **35** | 2 | 0 | 1 | 38 | 0.51 (0.25;0.76) |
|  | Somewhat important | 1 | **0** | 0 | 0 | 1 |  |
|  | Quite important | 3 | 0 | **3** | 1 | 7 |  |
|  | Very important | 3 | 1 | 1 | **2** | 7 |  |
|  | Total | 42 | 3 | 4 | 4 | **53** |  |

| Item | Test  Retest | Doesn’t apply/ not at all important | Somewhat important | Quite important | Very important | Total | Weighted Kappa (95% CI) |
| --- | --- | --- | --- | --- | --- | --- | --- |
| Hobbies | Doesn’t apply/ not at all important | **37** | 1 | 1 | 3 | 42 | 0.30 (0.03;0.57) |
|  | Somewhat important | 0 | **0** | 0 | 0 | 0 |  |
|  | Quite important | 2 | 0 | **1** | 1 | 4 |  |
|  | Very important | 4 | 0 | 0 | **2** | 6 |  |
|  | Total | 43 | 1 | 2 | 6 | **52** |  |
|  | | | | | | | |
| Driving | Doesn’t apply/ not at all important | **42** | 0 | 2 | 3 | 47 | 0.05 (nc) |
|  | Somewhat important | 1 | **1** | 0 | 0 | 2 |  |
|  | Quite important | 1 | 0 | **1** | 0 | 2 |  |
|  | Very important | 2 | 0 | 0 | **0** | 2 |  |
|  | Total | 46 | 1 | 3 | 3 | **53** |  |
|  | | | | | | | |
| Outings | Doesn’t apply/ not at all important | **34** | 0 | 2 | 2 | 38 | 0.28 (0.09;0.48) |
|  | Somewhat important | 1 | **0** | 1 | 0 | 2 |  |
|  | Quite important | 3 | 1 | **1** | 1 | 6 |  |
|  | Very important | 3 | 1 | 2 | **0** | 6 |  |
|  | Total | 41 | 2 | 6 | 3 | **52** |  |
|  | | | | | | | |
| Visiting | Doesn’t apply/ not at all important | **40** | 0 | 3 | 2 | 45 | 0.29 (0.05;0.53) |
|  | Somewhat important | 2 | **0** | 0 | 0 | 2 |  |
|  | Quite important | 1 | 0 | **0** | 1 | 2 |  |
|  | Very important | 2 | 0 | 2 | **0** | 4 |  |
|  | Total | 45 | 0 | 5 | 3 | **53** |  |
|  | | | | | | | |
| Home | Doesn’t apply/ not at all important | **49** | 0 | 0 | 2 | 51 | nc |
|  | Somewhat important | 0 | **0** | 0 | 0 | 0 |  |
|  | Quite important | 0 | 0 | **0** | 0 | 0 |  |
|  | Very important | 1 | 0 | 0 | **0** | 1 |  |
|  | Total | 50 | 0 | 0 | 2 | **52** |  |

| Item | Test  Retest | Doesn’t apply/ not at all important | Somewhat important | Quite important | Very important | Total | Weighted Kappa (95% CI) |
| --- | --- | --- | --- | --- | --- | --- | --- |
| Independence | Doesn’t apply/ not at all important | **40** | 0 | 1 | 2 | 43 | 0.54 (0.28;0.79) |
|  | Somewhat important | 0 | **0** | 0 | 0 | 0 |  |
|  | Quite important | 3 | 0 | **0** | 1 | 4 |  |
|  | Very important | 1 | 0 | 2 | **2** | 5 |  |
|  | Total | 44 | 0 | 3 | 5 | **52** |  |

**Table 2. Intra- and Inter-rater reliability**

| Item | Test  Retest | Doesn’t apply/ not at all important | Somewhat important | Quite important | Very important | Total | Weighted Kappa (95% CI) |
| --- | --- | --- | --- | --- | --- | --- | --- |
| Better  Intra-rater | Doesn’t apply/ not at all important | **3** | 0 | 0 | 2 | 5 | 0.62 (0.23;1) |
|  | Somewhat important | 0 | **0** | 0 | 0 | 0 |  |
|  | Quite important | 0 | 0 | **2** | 0 | 2 |  |
|  | Very important | 0 | 0 | 3 | **10** | 13 |  |
|  | Total | 3 | 0 | 5 | 12 | **20** |  |
|  | | | | | | | |
| Better  Inter-rater | Doesn’t apply/ not at all important | **0** | 0 | 0 | 8 | 8 | -0.16 (nc) |
|  | Somewhat important | 0 | **0** | 0 | 0 | 0 |  |
|  | Quite important | 1 | 1 | **4** | 3 | 9 |  |
|  | Very important | 2 | 0 | 1 | **13** | 16 |  |
|  | Total | 3 | 1 | 5 | 24 | **33** |  |
|  | | | | | | | |
| Energy  Intra-rater | Doesn’t apply/ not at all important | **7** | 0 | 0 | 0 | 7 | 0.62 (0.30;0.86) |
|  | Somewhat important | 0 | **0** | 0 | 0 | 0 |  |
|  | Quite important | 1 | 1 | **3** | 1 | 6 |  |
|  | Very important | 2 | 0 | 2 | **3** | 7 |  |
|  | Total | 10 | 1 | 5 | 4 | **20** |  |

| Item | Test  Retest | Doesn’t apply/ not at all important | Somewhat important | Quite important | Very important | Total | Weighted Kappa (95% CI) |
| --- | --- | --- | --- | --- | --- | --- | --- |
| Energy  Inter-rater | Doesn’t apply/ not at all important | **8** | 2 | 4 | 4 | 18 | 0.35 (0.09;0.61) |
|  | Somewhat important | 1 | **0** | 0 | 0 | 1 |  |
|  | Quite important | 1 | 0 | **4** | 2 | 7 |  |
|  | Very important | 1 | 0 | 2 | **4** | 7 |  |
|  | Total | 11 | 2 | 10 | 10 | **33** |  |
|  | | | | | | | |
| Pain  Intra-rater | Doesn’t apply/ not at all important | **13** | 0 | 0 | 1 | 14 | 0.56 (0.24;0.88) |
|  | Somewhat important | 0 | **0** | 0 | 0 | 0 |  |
|  | Quite important | 0 | 0 | **0** | 0 | 0 |  |
|  | Very important | 2 | 0 | 2 | **2** | 6 |  |
|  | Total | 15 | 0 | 2 | 3 | **20** |  |
|  | | | | | | | |
| Pain  Inter-rater | Doesn’t apply/ not at all important | **13** | 0 | 4 | 3 | 20 | 0.49 (0.24;0.74) |
|  | Somewhat important | 0 | **1** | 0 | 0 | 1 |  |
|  | Quite important | 2 | 0 | **0** | 2 | 4 |  |
|  | Very important | 0 | 2 | 0 | **6** | 8 |  |
|  | Total | 15 | 3 | 4 | 11 | **33** |  |
|  | | | | | | | |
| Bowel movement  Intra-rater | Doesn’t apply/ not at all important | **17** | 0 | 0 | 0 | 17 | 0.85 (0.51;1) |
|  | Somewhat important | 0 | **0** | 0 | 0 | 0 |  |
|  | Quite important | 1 | 0 | **1** | 0 | 2 |  |
|  | Very important | 0 | 0 | 0 | **1** | 1 |  |
|  | Total | 18 | 0 | 1 | 1 | **20** |  |
|  | | | | | | | |
| Bowel movement  Inter-rater | Doesn’t apply/ not at all important | **24** | 1 | 2 | 1 | 28 | 0.58 (0.21;0.95) |
|  | Somewhat important | 0 | **0** | 0 | 0 | 2 |  |
|  | Quite important | 0 | 0 | **2** | 0 | 2 |  |
|  | Very important | 1 | 0 | 0 | **2** | 3 |  |
|  | Total | 25 | 1 | 4 | 3 | **33** |  |

| Item | Test  Retest | | Doesn’t apply/ not at all important | | Somewhat important | | Quite important | | Very important | | Total | | Weighted Kappa (95% CI) | |
| --- | --- | --- | --- | --- | --- | --- | --- | --- | --- | --- | --- | --- | --- | --- |
| Shortness of breath  Intra-rater | Doesn’t apply/ not at all important | | **8** | | 0 | | 1 | | 1 | | 10 | | 0.56 (0.24;0.88) | |
|  | Somewhat important | | 0 | | **0** | | 0 | | 0 | | 0 | |  |  |
|  | Quite important | | 0 | | 0 | | **2** | | 2 | | 4 | |  |  |
|  | Very important | | 1 | | 1 | | 2 | | **2** | | 6 | |  |  |
|  | Total | | 9 | | 1 | | 5 | | 5 | | **20** | |  |  |
|  | | | | | | | | | | | | | | |
| Shortness of breath  Inter-rater | Doesn’t apply/ not at all important | | **11** | | 0 | | 1 | | 2 | | 14 | | 0.53 (0.26;0.80) | |
|  | Somewhat important | | 2 | | **1** | | 1 | | 1 | | 5 | |  |  |
|  | Quite important | | 1 | | 0 | | **2** | | 2 | | 5 | |  |  |
|  | Very important | | 2 | | 0 | | 1 | | **6** | | 9 | |  |  |
|  | Total | | 16 | | 1 | | 5 | | 11 | | **33** | |  |  |
|  | | | | | | | | | | | | | | |
| Walking  Intra-rater | | Doesn’t apply/ not at all important | | **9** | | 1 | | 0 | | 0 | | 10 | | 0.84 (0.72;0.97) |
|  |  | Somewhat important | | 1 | | **0** | | 0 | | 0 | | 1 | |  |
|  |  | Quite important | | 0 | | 0 | | **0** | | 0 | | 0 | |  |
|  |  | Very important | | 1 | | 0 | | 1 | | **6** | | 8 | |  |
|  |  | Total | | 11 | | 1 | | 1 | | 6 | | **19** | |  |
|  | | | | | | | | | | | | | | |
| Walking  Inter-rater | | Doesn’t apply/ not at all important | | **10** | | 0 | | 3 | | 3 | | 16 | | 0.49 (0.23;0.75) |
|  |  | Somewhat important | | 0 | | **0** | | 0 | | 0 | | 0 | |  |
|  |  | Quite important | | 1 | | 1 | | **1** | | 2 | | 5 | |  |
|  |  | Very important | | 1 | | 0 | | 3 | | **6** | | 10 | |  |
|  |  | Total | | 12 | | 1 | | 7 | | 11 | | **31** | |  |
|  | | | | | | | | | | | | | | |
| Appetite  Intra-rater | | Doesn’t apply/ not at all important | | **14** | | 0 | | 5 | | 0 | | 19 | | nc |
|  |  | Somewhat important | | 0 | | **0** | | 0 | | 0 | | 0 | |  |
|  |  | Quite important | | 0 | | **0** | | 0 | | 0 | | 0 | |  |
|  |  | Very important | | 0 | | **0** | | 0 | | 0 | | 0 | |  |
|  |  | Total | | 0 | | 0 | | 0 | | 0 | | **19** | |  |

| Item | Test  Retest | Doesn’t apply/ not at all important | Somewhat important | Quite important | Very important | Total | Weighted Kappa (95% CI) |
| --- | --- | --- | --- | --- | --- | --- | --- |
| Appetite  Inter-rater | Doesn’t apply/ not at all important | **24** | 2 | 1 | 0 | 27 | 0.46 (0;0.93) |
|  | Somewhat important | 2 | **0** | 0 | 0 | 2 |  |
|  | Quite important | 2 | 0 | **1** | 0 | 3 |  |
|  | Very important | 0 | 0 | 1 | **0** | 1 |  |
|  | Total | 28 | 2 | 3 | 0 | **33** |  |
|  | | | | | | | |
| Knowing what is wrong  Intra-rater | Doesn’t apply/ not at all important | **9** | 1 | 1 | 2 | 13 | 0.30 (0;0.68) |
|  | Somewhat important | 0 | **0** | 0 | 0 | 0 |  |
|  | Quite important | 1 | 0 | **1** | 0 | 2 |  |
|  | Very important | 2 | 0 | 1 | **2** | 5 |  |
|  | Total | 12 | 1 | 3 | 4 | **20** |  |
|  | | | | | | | |
| Knowing what is wrong  Inter-rater | Doesn’t apply/ not at all important | **19** | 1 | 1 | 1 | 22 | 0.58 (0.33;0.83) |
|  | Somewhat important | 0 | **0** | 0 | 0 | 0 |  |
|  | Quite important | 1 | 0 | **0** | 1 | 2 |  |
|  | Very important | 3 | 0 | 1 | **5** | 9 |  |
|  | Total | 23 | 1 | 2 | 7 | 33 |  |
|  | | | | | | | |
| Controlling disease  Intra-rater | Doesn’t apply/ not at all important | **7** | 0 | 0 | 4 | 11 | 0.50 (0.20;0.80) |
|  | Somewhat important | 0 | **0** | 0 | 0 | 0 |  |
|  | Quite important | 0 | 1 | **1** | 2 | 4 |  |
|  | Very important | 0 | 0 | 1 | **4** | 5 |  |
|  | Total | 7 | 1 | 2 | 10 | **20** |  |
|  | | | | | | | |
| Controlling disease  Inter-rater | Doesn’t apply/ not at all important | **8** | 0 | 2 | 4 | 14 | 0.38 (0.11;0.66) |
|  | Somewhat important | 1 | **0** | 1 | 0 | 2 |  |
|  | Quite important | 1 | 0 | **1** | 0 | 2 |  |
|  | Very important | 3 | 0 | 1 | **10** | 14 |  |
|  | Total | 13 | 0 | 5 | 14 | **32** |  |

| Item | Test  Retest | Doesn’t apply/ not at all important | Somewhat important | Quite important | Very important | Total | Weighted Kappa (95% CI) |
| --- | --- | --- | --- | --- | --- | --- | --- |
| Alive  Intra-rater | Doesn’t apply/ not at all important | **12** | 0 | 0 | 3 | 15 | 0.45 (0.09;0.80) |
|  | Somewhat important | 0 | **0** | 0 | 0 | 0 |  |
|  | Quite important | 0 | 0 | **1** | 1 | 2 |  |
|  | Very important | 1 | 0 | 0 | **2** | 3 |  |
|  | Total | 13 | 0 | 1 | 6 | **20** |  |
|  | | | | | | | |
| Alive  Inter-rater | Doesn’t apply/ not at all important | **10** | 0 | 1 | 6 | 17 | 0.16 (0;0.43) |
|  | Somewhat important | 0 | **0** | 0 | 0 | 0 |  |
|  | Quite important | 0 | 1 | **1** | 0 | 2 |  |
|  | Very important | 6 | 0 | 1 | **7** | 14 |  |
|  | Total | 16 | 1 | 3 | 13 | **33** |  |
|  | | | | | | | |
| Enjoying life  Intra-rater | Doesn’t apply/ not at all important | **15** | 0 | 0 | 2 | 17 | 0.65 (0.34;0.97) |
|  | Somewhat important | 0 | **0** | 0 | 0 | 0 |  |
|  | Quite important | 0 | 0 | **0** | 1 | 1 |  |
|  | Very important | 0 | 0 | 0 | **2** | 2 |  |
|  | Total | 15 | 0 | 0 | 5 | 20 |  |
|  | | | | | | | |
| Enjoying life  Inter-rater | Doesn’t apply/ not at all important | **16** | 0 | 2 | 6 | 24 | -0.12  (-0.39;  0.14) |
|  | Somewhat important | 0 | **0** | 0 | 0 | 0 |  |
|  | Quite important | 1 | 1 | **1** | 0 | 3 |  |
|  | Very important | 4 | 0 | 1 | **0** | 5 |  |
|  | Total | 21 | 1 | 4 | 6 | **32** |  |
|  | | | | | | | |
| Groceries  Intra-rater | Doesn’t apply/ not at all important | **18** | 0 | 0 | 0 | 18 | 0.85 (nc) |
|  | Somewhat important | 1 | **0** | 0 | 0 | 1 |  |
|  | Quite important | 0 | 0 | **0** | 0 | 0 |  |
|  | Very important | 0 | 0 | 1 | **0** | 1 |  |
|  | Total | 19 | 0 | 1 | 0 | **20** |  |

| Item | Test  Retest | Doesn’t apply/ not at all important | Somewhat important | Quite important | Very important | Total | Weighted Kappa (95% CI) |
| --- | --- | --- | --- | --- | --- | --- | --- |
| Groceries  Inter-rater | Doesn’t apply/ not at all important | **20** | 1 | 1 | 3 | 25 | 0.28 (0;0.57) |
|  | Somewhat important | 0 | **1** | 0 | 0 | 1 |  |
|  | Quite important | 0 | 0 | **1** | 1 | 2 |  |
|  | Very important | 3 | 0 | **1** | 1 | 5 |  |
|  | Total | 23 | 2 | 3 | 5 | **33** |  |
|  | | | | | | | |
| Washing and dressing  Intra-rater | Doesn’t apply/ not at all important | **14** | 0 | 0 | 3 | 17 | 0.29 (0;0.61) |
|  | Somewhat important | 0 | **0** | 0 | 0 | 0 |  |
|  | Quite important | 1 | 0 | **0** | 0 | 1 |  |
|  | Very important | 0 | 0 | 2 | **0** | 2 |  |
|  | Total | 15 | 0 | 2 | 3 | **20** |  |
|  | | | | | | | |
| Washing and dressing  Inter-rater | Doesn’t apply/ not at all important | **22** | 1 | 0 | 2 | 25 | 0.23 (0;0.53) |
|  | Somewhat important | 0 | **0** | 0 | 0 | 0 |  |
|  | Quite important | 1 | 0 | **1** | 0 | 2 |  |
|  | Very important | 3 | 1 | **0** | **1** | 5 |  |
|  | Total | 26 | 2 | 1 | 3 | **32** |  |
|  | | | | | | | |
| Gardening  Intra-rater | Doesn’t apply/ not at all important | **15** | 0 | 0 | 0 | 15 | 0.74 (0.41;1) |
|  | Somewhat important | 0 | **0** | 0 | 0 | 0 |  |
|  | Quite important | 0 | 1 | **1** | 0 | 2 |  |
|  | Very important | 1 | 0 | 1 | **1** | 3 |  |
|  | Total | 16 | 1 | 2 | 1 | **20** |  |
|  | | | | | | | |
| Gardening  Inter-rater | Doesn’t apply/ not at all important | **24** | 0 | 0 | 1 | 25 | 0.39 (0.05;0.74) |
|  | Somewhat important | 0 | **2** | 0 | 0 | 2 |  |
|  | Quite important | 1 | 0 | **2** | 0 | 3 |  |
|  | Very important | 2 | 0 | 1 | **0** | 3 |  |
|  | Total | 27 | 2 | 3 | 1 | **33** |  |

| Item | Test  Retest | Doesn’t apply/ not at all important | Somewhat important | Quite important | Very important | Total | Weighted Kappa (95% CI) |
| --- | --- | --- | --- | --- | --- | --- | --- |
| Sports  Intra-rater | Doesn’t apply/ not at all important | **13** | 1 | 0 | 1 | 15 | 0.65 (0.30;0.99) |
|  | Somewhat important | 0 | **0** | 0 | 0 | 0 |  |
|  | Quite important | 0 | 0 | **1** | 1 | 2 |  |
|  | Very important | 1 | 0 | 0 | **2** | 3 |  |
|  | Total | 14 | 1 | 1 | 4 | **20** |  |
|  | | | | | | | |
| Sports  Inter-rater | Doesn’t apply/ not at all important | **22** | 1 | 0 | 0 | 23 | 0.38 (0.10;0.66) |
|  | Somewhat important | 1 | **0** | 0 | 0 | 1 |  |
|  | Quite important | 3 | 0 | **2** | 0 | 5 |  |
|  | Very important | 2 | 1 | 1 | **0** | 4 |  |
|  | Total | 28 | 2 | 3 | 0 | **33** |  |
|  | | | | | | | |
| Hobbies  Intra-rater | Doesn’t apply/ not at all important | **13** | 1 | 0 | 2 | 16 | 0.34 (0;0.74) |
|  | Somewhat important | 0 | **0** | 0 | 0 | 0 |  |
|  | Quite important | 1 | 0 | **0** | 1 | 2 |  |
|  | Very important | 1 | 0 | 0 | **1** | 2 |  |
|  | Total | 15 | 1 | 0 | 4 | **20** |  |
|  | | | | | | | |
| Hobbies  Inter-rater | Doesn’t apply/ not at all important | **24** | 0 | 1 | 1 | 26 | 0.27 (0;0.62) |
|  | Somewhat important | 0 | **0** | 0 | 0 | 0 |  |
|  | Quite important | 1 | 0 | **1** | 0 | 2 |  |
|  | Very important | 3 | 0 | 0 | **1** | 4 |  |
|  | Total | 28 | 0 | 2 | 2 | **32** |  |
|  | | | | | | | |
| Drive  Intra-rater | Doesn’t apply/ not at all important | **18** | 0 | 0 | 0 | 18 | 0.44 (0;1) |
|  | Somewhat important | 0 | **0** | 0 | 0 | 0 |  |
|  | Quite important | 0 | 0 | **1** | 0 | 1 |  |
|  | Very important | 1 | 0 | 0 | **0** | 1 |  |
|  | Total | 19 | 0 | 1 | 0 | **20** |  |
|  | | | | | | | |
| Item | Test  Retest | Doesn’t apply/ not at all important | Somewhat important | Quite important | Very important | Total | Weighted Kappa (95% CI) |
| Drive  Inter-rater | Doesn’t apply/ not at all important | **24** | 0 | 2 | 3 | 29 | -0.08 (nc) |
|  | Somewhat important | 1 | **1** | 0 | 0 | 2 |  |
|  | Quite important | 1 | 0 | **0** | 0 | 1 |  |
|  | Very important | 1 | 0 | 0 | **0** | 1 |  |
|  | Total | 27 | 1 | 2 | 3 | **33** |  |
|  | | | | | | | |
| Outings  Intra-rater | Doesn’t apply/ not at all important | **14** | 0 | 0 | 0 | 14 | 0.44 (0.13;0.74) |
|  | Somewhat important | 1 | **0** | 1 | 0 | 2 |  |
|  | Quite important | 1 | 0 | **0** | 0 | 1 |  |
|  | Very important | 1 | 1 | 1 | **0** | 3 |  |
|  | Total | 17 | 1 | 2 | 0 | **20** |  |
|  | | | | | | | |
| Outings  Inter-rater | Doesn’t apply/ not at all important | **20** | 0 | 2 | 2 | 24 | 0.21 (0;0.43) |
|  | Somewhat important | 0 | **0** | 0 | 0 | 0 |  |
|  | Quite important | 2 | 1 | **1** | 1 | 5 |  |
|  | Very important | 2 | 0 | 1 | **0** | 3 |  |
|  | Total | 24 | 1 | 4 | 3 | **32** |  |
|  | | | | | | | |
| Visiting  Intra-rater | Doesn’t apply/ not at all important | **15** | 0 | 1 | 1 | 17 | 0.47 (0.09;0.85) |
|  | Somewhat important | 0 | **0** | 0 | 0 | 0 |  |
|  | Quite important | 1 | 0 | **0** | 0 | 1 |  |
|  | Very important | 0 | 0 | 2 | **0** | 2 |  |
|  | Total | 16 | 0 | 3 | 1 | **20** |  |
|  | | | | | | | |
| Visiting  Inter-raters | Doesn’t apply/ not at all important | **25** | 0 | 2 | 1 | 28 | 0.14 (0;0.28) |
|  | Somewhat important | 2 | **0** | 0 | 0 | 2 |  |
|  | Quite important | 0 | 0 | **0** | 1 | 1 |  |
|  | Very important | 2 | 0 | 0 | **0** | 2 |  |
|  | Total | 29 | 0 | 2 | 2 | **33** |  |

| Item | Test  Retest | Doesn’t apply/ not at all important | Somewhat important | Quite important | Very important | Total | Weighted Kappa (95% CI) |
| --- | --- | --- | --- | --- | --- | --- | --- |
| Home  Intra-rater | Doesn’t apply/ not at all important | **19** | 0 | 0 | 0 | 19 | nc |
|  | Somewhat important | 0 | 0 | 0 | 0 | 0 |  |
|  | Quite important | 0 | 0 | 0 | 0 | 0 |  |
|  | Very important | 1 | 0 | 0 | 0 | 1 |  |
|  | Total | 20 | 0 | 0 | 0 | **20** |  |
|  | | | | | | | |
| Home  Inter-rater | Doesn’t apply/ not at all important | **30** | 0 | 0 | 2 | 32 | nc |
|  | Somewhat important | 0 | 0 | 0 | 0 | 0 |  |
|  | Quite important | 0 | 0 | 0 | 0 | 0 |  |
|  | Very important | 0 | 0 | 0 | 0 | 0 |  |
|  | Total | 30 | 0 | 0 | 2 | **32** |  |
|  | | | | | | | |
| Independence  Intra-rater | Doesn’t apply/ not at all important | **16** | 0 | 0 | 0 | 16 | 0.74 (0.48;0.99) |
|  | Somewhat important | 0 | **0** | 0 | 0 | 0 |  |
|  | Quite important | 2 | 0 | **0** | 0 | 2 |  |
|  | Very important | 0 | 0 | 1 | **1** | 2 |  |
|  | Total | 18 | 0 | 1 | 1 | **20** |  |
|  | | | | | | | |
| Independence  Inter-raters | Doesn’t apply/ not at all important | **24** | 0 | 1 | 2 | 27 | 0.44 (0.11;0.77) |
|  | Somewhat important | 0 | **0** | 0 | 0 | 0 |  |
|  | Quite important | 1 | 0 | **0** | 1 | 2 |  |
|  | Very important | 1 | 0 | 1 | **1** | 3 |  |
|  | Total | 26 | 0 | 2 | 4 | **32** |  |
