# Supplementary material for: Reliability and validity of the Patient Benefit Assessment Scale for Hospitalised Older Patients (P-BAS HOP)
Source: BMC Geriatr. 2021 Mar 1;21:149. doi: 10.1186/s12877-021-02079-z (PMC7923656; doi:10.1186/s12877-021-02079-z)
Supplement: Supplementary file 4 — Additional file 4. [file 12877_2021_2079_MOESM4_ESM.docx]

**Additional file 4. Crosstabulations of test-retest Follow-up**

**Reliability and validity of the Patient Benefit Assessment Scale for Hospitalised Older Patients (P-BAS HOP)**

**Authors:**

1. Maria Johanna van der Kluit, MSc RN (Corresponding author)

University of Groningen, University Medical Center Groningen, University Center for Geriatric Medicine, Hanzeplein 1, 9700 RB Groningen, The Netherlands

[m.j.van.der.kluit@umcg.nl](mailto:m.j.van.der.kluit@umcg.nl)

+31503613921

1. Geke J. Dijkstra, PhD

University of Groningen, University Medical Center Groningen, Department of Health Sciences, Applied Health Research, Groningen, The Netherlands

NHL Stenden University of Applied Sciences, Research Group Living, Wellbeing and Care for Older People, Leeuwarden, The Netherlands

[g.j.dijkstra@umcg.nl](mailto:g.j.dijkstra@umcg.nl)

1. Sophia E. de Rooij, MD PhD

University of Groningen, University Medical Center Groningen, University Center for Geriatric Medicine, Groningen, The Netherlands

Medical Spectrum Twente, Medical School Twente, Enschede, The Netherlands

sejaderooij@gmail.com

**Additional file 4. Crosstabulations of test-retest Follow-up. Table 1 Overall test-retest**

Retest

| Item | Test | Not at all | Somewhat | Quite | Completely | Total | Weighted Kappa (95% CI) |
| --- | --- | --- | --- | --- | --- | --- | --- |
| Better | Not at all | **2** | 2 | 1 | 1 | 6 | 0.51 (0.20;0.81) |
|  | Somewhat | 1 | **4** | 8 | 1 | 14 |  |
|  | Quite | 0 | 3 | **12** | 4 | 19 |  |
|  | Completely | 0 | 1 | 3 | **8** | 12 |  |
|  | Total | 3 | 10 | 24 | 14 | **51** |  |
|  | | | | | | | |
| Energy | Not at all | **3** | 5 | 2 | 1 | 11 | 0.45 (0.16;0.74) |
|  | Somewhat | 4 | **5** | 4 | 0 | 13 |  |
|  | Quite | 1 | 1 | **6** | 2 | 10 |  |
|  | Completely | 1 | 0 | 2 | **4** | 7 |  |
|  | Total | 9 | 11 | 14 | 7 | **41** |  |
|  | | | | | | | |
| Pain | Not at all | **3** | 1 | 0 | 0 | 4 | 0.72 (nc) |
|  | Somewhat | 1 | **0** | 0 | 0 | 1 |  |
|  | Quite | 1 | 2 | **1** | 2 | 6 |  |
|  | Completely | 0 | 0 | 1 | **2** | 3 |  |
|  | Total | 5 | 3 | 2 | 4 | **14** |  |
|  | | | | | | | |
| Bowel movement | Not at all | **0** | 1 | 0 | 0 | 1 | nc |
|  | Somewhat | 0 | **0** | 0 | 1 | 1 |  |
|  | Quite | 0 | 0 | **0** | 0 | 0 |  |
|  | Completely | 0 | 0 | 1 | **2** | 3 |  |
|  | Total | 0 | 1 | 1 | 3 | **5** |  |
|  | | | | | | | |
| Shortness of breath | Not at all | **4** | 0 | 0 | 2 | 6 | 0.47 (0.07;0.88) |
|  | Somewhat | 3 | **1** | 3 | 1 | 8 |  |
|  | Quite | 0 | 2 | **6** | 0 | 8 |  |
|  | Completely | 0 | 0 | 0 | **3** | 3 |  |
|  | Total | 7 | 3 | 9 | 6 | **25** |  |
|  | | | | | | | |
| Walking | Not at all | **5** | 0 | 0 | 3 | 8 | 0.24 (0;0.50) |
|  | Somewhat | 2 | **1** | 3 | 1 | 7 |  |
|  | Quite | 0 | 2 | **1** | 2 | 5 |  |
|  | Completely | 1 | 1 | 1 | **2** | 5 |  |
|  | Total | 8 | 4 | 5 | 8 | **25** |  |
|  | | | | | | | |
| Appetite | Not at all | **2** | 1 | 0 | 1 | 4 | nc |
|  | Somewhat | 0 | **2** | 0 | 0 | 2 |  |
|  | Quite | 0 | 0 | **1** | 0 | 1 |  |
|  | Completely | 0 | 0 | 0 | **2** | 2 |  |
|  | Total | 2 | 3 | 1 | 3 | **9** |  |
|  | | | | | | | |
| Knowing what is wrong | Not at all | **0** | 0 | 1 | 0 | 1 | 0.17 (nc) |
|  | Somewhat | 0 | **0** | 0 | 0 | 0 |  |
|  | Quite | 0 | 0 | **0** | 1 | 1 |  |
|  | Completely | 1 | 0 | 0 | **7** | 8 |  |
|  | Total | 1 | 0 | 1 | 8 | **10** |  |
|  | | | | | | | |
| Controlling disease | Not at all | **4** | 0 | 1 | 1 | 6 | 0.59 (0.28;0.90) |
|  | Somewhat | 0 | **2** | 1 | 0 | 3 |  |
|  | Quite | 0 | 4 | **1** | 1 | 6 |  |
|  | Completely | 1 | 0 | 4 | **9** | 14 |  |
|  | Total | 5 | 6 | 7 | 11 | **29** |  |
|  | | | | | | | |
| Alive | Not at all | **1** | 1 | 1 | 1 | 4 | 0.50 (0.18;0.82) |
|  | Somewhat | 0 | **0** | 2 | 2 | 4 |  |
|  | Quite | 0 | 0 | **1** | 2 | 3 |  |
|  | Completely | 0 | 0 | 2 | **15** | 17 |  |
|  | Total | 1 | 1 | 6 | 20 | **28** |  |
| Item | Test  Retest | Not at all | Somewhat | Quite | Completely | Total | Weighted Kappa (95% CI) |
| Enjoying life | Not at all | **3** | 0 | 0 | 0 | 3 | 0.88 (0.65;1) |
|  | Somewhat | 0 | **2** | 1 | 0 | 3 |  |
|  | Quite | 0 | 0 | **3** | 0 | 3 |  |
|  | Completely | 0 | 1 | 0 | **7** | 8 |  |
|  | Total | 3 | 3 | 4 | 7 | **17** |  |
|  | | | | | | | |
| Groceries | Not at all | **0** | 1 | 0 | 1 | 2 | nc |
|  | Somewhat | 0 | **0** | 1 | 0 | 1 |  |
|  | Quite | 1 | 0 | **0** | 0 | 1 |  |
|  | Completely | 0 | 1 | 0 | **0** | 1 |  |
|  | Total | 1 | 2 | 1 | 1 | **5** |  |
|  | | | | | | | |
| Washing and dressing | Not at all | **0** | 0 | 0 | 0 | 0 | nc |
|  | Somewhat | 0 | **0** | 0 | 0 | 0 |  |
|  | Quite | 0 | 1 | **0** | 0 | 1 |  |
|  | Completely | 0 | 0 | 1 | **3** | 4 |  |
|  | Total | 0 | 1 | 1 | 3 | **5** |  |
|  | | | | | | | |
| Gardening | Not at all | **2** | 0 | 0 | 1 | 3 | 0.40 (0;87) |
|  | Somewhat | 0 | **1** | 2 | 1 | 4 |  |
|  | Quite | 1 | 0 | **1** | 1 | 3 |  |
|  | Completely | 0 | 0 | 1 | **2** | 3 |  |
|  | Total | 3 | 1 | 4 | 5 | **13** |  |
|  | | | | | | | |
| Sports | Not at all | **4** | 1 | 1 | 0 | 6 | 0.61 (0.39;0.83) |
|  | Somewhat | 0 | **0** | 0 | 2 | 2 |  |
|  | Quite | 0 | 0 | **0** | 1 | 1 |  |
|  | Completely | 0 | 0 | 2 | **1** | 3 |  |
|  | Total | 4 | 1 | 3 | 4 | **12** |  |
|  | | | | | | | |
| Hobbies | Not at all | **2** | 1 | 0 | 0 | 3 | nc |
|  | Somewhat | 1 | **1** | 0 | 0 | 2 |  |
|  | Quite | 0 | 0 | **0** | 0 | 0 |  |
|  | Completely | 0 | 1 | 0 | **0** | 1 |  |
|  | Total | 3 | 3 | 0 | 0 | **6** |  |
|  | | | | | | | |
| Driving | Not at all | **4** | 1 | 0 | 1 | 6 | 0.55 (0.07;1) |
|  | Somewhat | 1 | **0** | 0 | 0 | 1 |  |
|  | Quite | 1 | 0 | **0** | 0 | 1 |  |
|  | Completely | 0 | 0 | 0 | **2** | 2 |  |
|  | Total | 6 | 1 | 0 | 3 | **10** |  |
|  | | | | | | | |
| Outings | Not at all | **4** | 1 | 0 | 0 | 5 | nc |
|  | Somewhat | 1 | **0** | 1 | 0 | 2 |  |
|  | Quite | 0 | 0 | **0** | 0 | 0 |  |
|  | Completely | 0 | 0 | 0 | **0** | 0 |  |
|  | Total | 5 | 1 | 1 | 0 | **7** |  |
|  | | | | | | | |
| Visiting | Not at all | **1** | 0 | 0 | 0 | 1 | nc |
|  | Somewhat | 2 | **0** | 1 | 0 | 3 |  |
|  | Quite | 0 | 0 | **0** | 0 | 0 |  |
|  | Completely | 0 | 0 | 0 | **0** | 0 |  |
|  | Total | 3 | 0 | 1 | 0 | **4** |  |
|  | | | | | | | |
| Home | Not at all | **1** | 0 | 0 | 0 | 1 | nc |
|  | Somewhat | 0 | **0** | 0 | 0 | 0 |  |
|  | Quite | 0 | 0 | **0** | 0 | 0 |  |
|  | Completely | 0 | 0 | 0 | **0** | 0 |  |
|  | Total | 1 | 0 | 0 | 0 | **1** |  |

| Item | Test  Retest | Not at all | Somewhat | Quite | Completely | Total | Weighted Kappa (95% CI) |
| --- | --- | --- | --- | --- | --- | --- | --- |
| Independence | Not at all | **1** | 0 | 0 | 0 | 1 | nc |
|  | Somewhat | 1 | **0** | 0 | 1 | 2 |  |
|  | Quite | 2 | 0 | **0** | 1 | 3 |  |
|  | Completely | 0 | 0 | 1 | **0** | 1 |  |
|  | Total | 4 | 0 | 1 | 2 | **7** |  |
|  | | | | | | | |
| Extra | Not at all | **0** | 0 | 0 | 0 | 0 | nc |
|  | Somewhat | 0 | **1** | 0 | 0 | 1 |  |
|  | Quite | 0 | 0 | **0** | 0 | 0 |  |
|  | Completely | 0 | 0 | 0 | **1** | 1 |  |
|  | Total | 0 | 1 | 0 | 1 | **2** |  |

**Table 2. Intra- and Inter-rater reliability**

Retest

| Item | Test | Not at all | Somewhat | Quite | | Completely | Total | Weighted Kappa (95% CI) |
| --- | --- | --- | --- | --- | --- | --- | --- | --- |
| Better  Intra-rater | not at all | **1** | 1 | 0 | | 0 | 2 | 0.59 (0.21;0.98) |
|  | Somewhat | 0 | **2** | 3 | | 1 | 6 |  |
|  | Quite | 0 | 3 | **3** | | 3 | 9 |  |
|  | Completely | 0 | 0 | 1 | | **4** | 5 |  |
|  | Total | 1 | 6 | 7 | | 8 | **22** |  |
|  | | | | |  | | | |
| Better  Inter-rater | not at all | **1** | 1 | 1 | | 1 | 4 | 0.43 (0.05;0.82) |
|  | Somewhat | 1 | **2** | 5 | | 0 | 8 |  |
|  | Quite | 0 | 0 | **9** | | 1 | 10 |  |
|  | Completely | 0 | 1 | 2 | | **4** | 7 |  |
|  | Total | 2 | 4 | 17 | | 6 | **29** |  |
|  | | | | | | | | |
| Energy  Intra-rater | not at all | **1** | 3 | 1 | | 0 | 5 | 0.47 (0.05;0.89) |
|  | Somewhat | 2 | **3** | 2 | | 0 | 7 |  |
|  | Quite | 1 | 0 | **4** | | 1 | 6 |  |
|  | Completely | 1 | 0 | 0 | | **3** | 4 |  |
|  | Total | 5 | 6 | 7 | | 4 | **22** |  |
|  | | | | | | | | |
| Energy  Inter-rater | not at all | **2** | 2 | 1 | | 1 | 6 | 0.44 (0.06;0.82) |
|  | Somewhat | 2 | **2** | 2 | | 0 | 6 |  |
|  | Quite | 0 | 1 | **2** | | 1 | 4 |  |
|  | Completely | 0 | 0 | 2 | | **1** | 3 |  |
|  | Total | 4 | 5 | 7 | | 3 | **19** |  |
|  | | | | | | | | |
| Pain  Intra-rater | not at all | **1** | 0 | 0 | | 0 | 1 | nc |
|  | Somewhat | 0 | **0** | 0 | | 0 | 0 |  |
|  | Quite | 0 | 1 | **0** | | 0 | 1 |  |
|  | Completely | 0 | 0 | 1 | | **2** | 3 |  |
|  | Total | 1 | 1 | 1 | | 2 | **5** |  |
|  | | | | | | | | |
| Pain  Inter-rater | not at all | **2** | 1 | 0 | | 0 | 3 | nc |
|  | Somewhat | 1 | **0** | 0 | | 0 | 1 |  |
|  | Quite | 1 | 1 | **1** | | 2 | 5 |  |
|  | Completely | 0 | 0 | 0 | | **0** | 0 |  |
|  | Total | 4 | 2 | 1 | | 2 | **9** |  |
|  | | | | | | | | |
| Bowel movement  Intra-rater | not at all | **0** | 0 | 0 | | 0 | 0 | nc |
|  | Somewhat | 0 | **0** | 0 | | 0 | 0 |  |
|  | Quite | 0 | 0 | **0** | | 0 | 0 |  |
|  | Completely | 0 | 0 | 1 | | **2** | 3 |  |
|  | Total | 0 | 0 | 1 | | 2 | **3** |  |

| Item | Test  Retest | | Not at all | | Somewhat | | Quite | | Completely | | Total | | Weighted Kappa (95% CI) |
| --- | --- | --- | --- | --- | --- | --- | --- | --- | --- | --- | --- | --- | --- |
| Bowel movement  Inter-rater | not at all | | **0** | | 1 | | 0 | | 0 | | 1 | | nc |
|  | Somewhat | | 0 | | **0** | | 0 | | 1 | | 1 | |  |
|  | Quite | | 0 | | 0 | | **0** | | 0 | | 0 | |  |
|  | Completely | | 0 | | 0 | | 0 | | **0** | | 0 | |  |
|  | Total | | 0 | | 1 | | 0 | | 1 | | **2** | |  |
|  | | | | | | | | | | | | | |
| Shortness of breath  Intra-rater | not at all | | **3** | | 0 | | 0 | | 2 | | 5 | | 0.34 (0;0.78) |
|  | Somewhat | | 2 | | **0** | | 1 | | 0 | | 3 | |  |
|  | Quite | | 0 | | 1 | | **3** | | 0 | | 4 | |  |
|  | Completely | | 0 | | 0 | | 0 | | **1** | | 1 | |  |
|  | Total | | 5 | | 1 | | 4 | | 3 | | **13** | |  |
|  | | | | | | | | | | | | | |
| Shortness of breath  Inter-rater | not at all | | **1** | | 0 | | 0 | | 0 | | 1 | | 0.63 (0.03;1) |
|  | Somewhat | | 1 | | **1** | | 2 | | 1 | | 5 | |  |
|  | Quite | | 0 | | 1 | | **3** | | 0 | | 4 | |  |
|  | Completely | | 0 | | 0 | | 0 | | **2** | | 2 | |  |
|  | Total | | 2 | | 2 | | 5 | | 3 | | **12** | |  |
|  | | | | | | | | | | | | | |
| Walking  Intra-rater | | not at all | | **2** | | 0 | | 0 | | 2 | | 4 | 0.04 (nc) |
|  |  | Somewhat | | 1 | | **1** | | 2 | | 0 | | 4 |  |
|  |  | Quite | | 0 | | 0 | | **1** | | 0 | | 1 |  |
|  |  | Completely | | 1 | | 1 | | 1 | | **1** | | 4 |  |
|  |  | Total | | 4 | | 2 | | 4 | | 3 | | **13** |  |
|  | | | | | | | | | | | | | |
| Walking  Inter-rater | | not at all | | **3** | | 0 | | 0 | | 1 | | 4 | 0.45 (0.04;0.87) |
|  |  | Somewhat | | 1 | | **0** | | 1 | | 1 | | 3 |  |
|  |  | Quite | | 0 | | 2 | | **0** | | 2 | | 4 |  |
|  |  | Completely | | 0 | | 0 | | 0 | | **1** | | 1 |  |
|  |  | Total | | 4 | | 2 | | 1 | | 5 | | **12** |  |
|  | | | | | | | | | | | | | |
| Appetite  Intra-rater | | not at all | | **2** | | 0 | | 0 | | 0 | | 2 | nc |
|  |  | Somewhat | | 0 | | **2** | | 0 | | 0 | | 2 |  |
|  |  | Quite | | 0 | | **0** | | 0 | | 0 | | 0 |  |
|  |  | Completely | | 0 | | **0** | | 0 | | **2** | | 2 |  |
|  |  | Total | | 2 | | 2 | | 0 | | 2 | | **6** |  |
|  | | | | | | | | | | | | | |
| Appetite  Inter-rater | | not at all | | **0** | | 1 | | 0 | | 1 | | 2 | nc |
|  |  | Somewhat | | 0 | | **0** | | 0 | | 0 | | 0 |  |
|  |  | Quite | | 0 | | 0 | | **1** | | 0 | | 1 |  |
|  |  | Completely | | 0 | | 0 | | 0 | | **0** | | 0 |  |
|  |  | Total | | 0 | | 1 | | 1 | | 1 | | **3** |  |
|  | | | | | | | | | | | | | |
| Knowing what is wrong  Intra-rater | | not at all | | **0** | | 0 | | 0 | | 0 | | 0 | nc |
|  |  | Somewhat | | 0 | | **0** | | 0 | | 0 | | 0 |  |
|  |  | Quite | | 0 | | 0 | | **0** | | 0 | | 0 |  |
|  |  | Completely | | 1 | | 0 | | 0 | | **1** | | 2 |  |
|  |  | Total | | 1 | | 0 | | 0 | | 1 | | **2** |  |
|  | | | | | | | | | | | | | |
| Knowing what is wrong  Inter-rater | | not at all | | **0** | | 0 | | 1 | | 0 | | 1 | nc |
|  |  | Somewhat | | 0 | | **0** | | 0 | | 0 | | 0 |  |
|  |  | Quite | | 0 | | 0 | | **0** | | 1 | | 1 |  |
|  |  | Completely | | 0 | | 0 | | 0 | | **6** | | 6 |  |
|  |  | Total | | 0 | | 0 | | 1 | | 7 | | 8 |  |
|  | | | | | | | | | | | | | |
| Controlling disease  Intra-rater | | not at all | | **1** | | 0 | | 0 | | 1 | | 2 | 0.36 (0;0.88) |
|  |  | Somewhat | | 0 | | **1** | | 0 | | 0 | | 1 |  |
|  |  | Quite | | 0 | | 1 | | **0** | | 0 | | 1 |  |
|  |  | Completely | | 1 | | 0 | | 2 | | **5** | | 8 |  |
|  |  | Total | | 2 | | 2 | | 2 | | 6 | | **12** |  |

| Item | Test  Retest | Not at all | Somewhat | Quite | Completely | Total | Weighted Kappa (95% CI) |
| --- | --- | --- | --- | --- | --- | --- | --- |
| Controlling disease  Inter-rater | not at all | **3** | 0 | 1 | 0 | 4 | 0.74 (0.59;0.90) |
|  | Somewhat | 0 | **1** | 1 | 0 | 2 |  |
|  | Quite | 0 | 3 | **1** | 1 | 5 |  |
|  | Completely | 0 | 0 | 2 | **4** | 6 |  |
|  | Total | 3 | 4 | 5 | 5 | **17** |  |
|  | | | | | | | |
| Alive  Intra-rater | not at all | **1** | 1 | 0 | 1 | 3 | 0.57 (0.17;0.97) |
|  | Somewhat | 0 | **0** | 0 | 1 | 1 |  |
|  | Quite | 0 | 0 | **0** | 0 | 0 |  |
|  | Completely | 0 | 0 | 1 | **8** | 9 |  |
|  | Total | 1 | 1 | 1 | 10 | **13** |  |
|  | | | | | | | |
| Alive  Inter-rater | not at all | **0** | 0 | 1 | 0 | 1 | 0.38 (0.11;0.65) |
|  | Somewhat | 0 | **0** | 2 | 1 | 3 |  |
|  | Quite | 0 | 0 | **1** | 2 | 3 |  |
|  | Completely | 0 | 0 | 1 | **7** | 8 |  |
|  | Total | 0 | 0 | 5 | 10 | **15** |  |
|  | | | | | | | |
| Enjoying life  Intra-rater | not at all | **1** | 0 | 0 | 0 | 1 | nc |
|  | Somewhat | 0 | **1** | 1 | 0 | 2 |  |
|  | Quite | 0 | 0 | **1** | 0 | 1 |  |
|  | Completely | 0 | 0 | 0 | **3** | 3 |  |
|  | Total | 1 | 1 | 2 | 3 | **7** |  |
|  | | | | | | | |
| Enjoying life  Inter-rater | not at all | **2** | 0 | 0 | 0 | 2 | nc |
|  | Somewhat | 0 | **1** | 0 | 0 | 1 |  |
|  | Quite | 0 | 0 | **2** | 0 | 2 |  |
|  | Completely | 0 | 1 | 0 | **4** | 5 |  |
|  | Total | 2 | 2 | 2 | 4 | **10** |  |
|  | | | | | | | |
| Groceries  Intra-rater | not at all | **0** | 1 | 0 | 1 | 2 | nc |
|  | Somewhat | 0 | **0** | 1 | 0 | 1 |  |
|  | Quite | 1 | 0 | **0** | 0 | 1 |  |
|  | Completely | 0 | 0 | 0 | **0** | 0 |  |
|  | Total | 1 | 1 | 1 | 1 | **4** |  |
|  | | | | | | | |
| Groceries  Inter-rater | not at all | **0** | 0 | 0 | 0 | 0 | nc |
|  | Somewhat | 0 | **0** | 0 | 0 | 0 |  |
|  | Quite | 0 | 0 | **0** | 0 | 0 |  |
|  | Completely | 0 | 1 | **0** | 0 | 1 |  |
|  | Total | 0 | 1 | 0 | 0 | **1** |  |
|  | | | | | | | |
| Washing and dressing  Intra-rater | not at all | **0** | 0 | 0 | 0 | 0 | nc |
|  | Somewhat | 0 | **0** | 0 | 0 | 0 |  |
|  | Quite | 0 | 1 | **0** | 0 | 1 |  |
|  | Completely | 0 | 0 | 0 | **2** | 2 |  |
|  | Total | 0 | 1 | 0 | 2 | **3** |  |
|  | | | | | | | |
| Washing and dressing  Inter-rater | not at all | **0** | 0 | 0 | 0 | 0 | nc |
|  | Somewhat | 0 | **0** | 0 | 0 | 0 |  |
|  | Quite | 0 | 0 | **0** | 0 | 0 |  |
|  | Completely | 0 | 0 | **1** | **1** | 2 |  |
|  | Total | 0 | 0 | 1 | 1 | 2 |  |
|  | | | | | | | |
| Gardening  Intra-rater | not at all | **0** | 0 | 0 | 0 | 0 | nc |
|  | Somewhat | 0 | **0** | 1 | 1 | 1 |  |
|  | Quite | 1 | 0 | **1** | 0 | 2 |  |
|  | Completely | 0 | 0 | 0 | **2** | 2 |  |
|  | Total | 1 | 0 | 2 | 3 | **6** |  |

| Item | Test  Retest | Not at all | Somewhat | Quite | Completely | Total | Weighted Kappa (95% CI) |
| --- | --- | --- | --- | --- | --- | --- | --- |
| Gardening  Inter-rater | not at all | **2** | 0 | 0 | 1 | 3 | nc |
|  | Somewhat | 0 | **1** | 1 | 0 | 2 |  |
|  | Quite | 0 | 0 | **0** | 1 | 1 |  |
|  | Completely | 0 | 0 | 1 | **0** | 1 |  |
|  | Total | 2 | 1 | 2 | 2 | **7** |  |
|  | | | | | | | |
| Sports  Intra-rater | not at all | **2** | 0 | 0 | 0 | 2 | nc |
|  | Somewhat | 0 | **0** | 0 | 1 | 1 |  |
|  | Quite | 0 | 0 | **0** | 0 | 0 |  |
|  | Completely | 0 | 0 | 2 | **1** | 3 |  |
|  | Total | 2 | 0 | 2 | 2 | **6** |  |
|  | | | | | | | |
| Sports  Inter-rater | not at all | **2** | 1 | 1 | 0 | 4 | nc |
|  | Somewhat | 0 | **0** | 0 | 1 | 1 |  |
|  | Quite | 0 | 0 | **0** | 1 | 1 |  |
|  | Completely | 0 | 0 | 0 | **0** | 0 |  |
|  | Total | 2 | 1 | 1 | 2 | **6** |  |
|  | | | | | | | |
| Hobbies  Intra-rater | not at all | **1** | 0 | 0 | 0 | 1 | nc |
|  | Somewhat | 0 | **1** | 0 | 0 | 1 |  |
|  | Quite | 0 | 0 | **0** | 0 | 0 |  |
|  | Completely | 0 | 0 | 0 | **0** | 0 |  |
|  | Total | 1 | 1 | 0 | 0 | 2 |  |
|  | | | | | | | |
| Hobbies  Inter-rater | not at all | **1** | 1 | 0 | 0 | 2 | nc |
|  | Somewhat | 1 | **0** | 0 | 0 | 1 |  |
|  | Quite | 0 | 0 | **0** | 0 | 0 |  |
|  | Completely | 0 | 1 | 0 | **0** | 1 |  |
|  | Total | 2 | 2 | 0 | 0 | **4** |  |
|  | | | | | | | |
| Drive  Intra-rater | not at all | **2** | 0 | 0 | 0 | 2 | nc |
|  | Somewhat | 0 | **0** | 0 | 0 | 0 |  |
|  | Quite | 0 | 0 | **0** | 0 | 0 |  |
|  | Completely | 0 | 0 | 0 | **1** | 1 |  |
|  | Total | 2 | 0 | 0 | 1 | **3** |  |
|  | | | | | | | |
| Drive  Inter-rater | not at all | **2** | 1 | 0 | 1 | 4 | nc |
|  | Somewhat | 1 | **0** | 0 | 0 | 1 |  |
|  | Quite | 1 | 0 | **0** | 0 | 1 |  |
|  | Completely | 0 | 0 | 0 | **1** | 1 |  |
|  | Total | 4 | 1 | 0 | 2 | **7** |  |
|  | | | | | | | |
| Outings  Intra-rater | not at all | **3** | 0 | 0 | 0 | 3 | nc |
|  | Somewhat | 1 | **0** | 0 | 0 | 1 |  |
|  | Quite | 0 | 0 | **0** | 0 | 0 |  |
|  | Completely | 0 | 0 | 0 | **0** | 0 |  |
|  | Total | 4 | 0 | 0 | 0 | **4** |  |
|  | | | | | | | |
| Outings  Inter-rater | not at all | **1** | 1 | 0 | 0 | 2 | nc |
|  | Somewhat | 0 | **0** | 1 | 0 | 1 |  |
|  | Quite | 0 | 0 | **0** | 0 | 0 |  |
|  | Completely | 0 | 0 | 0 | **0** | 0 |  |
|  | Total | 1 | 1 | 1 | 0 | **3** |  |
|  | | | | | | | |
| Visiting  Intra-rater | not at all | **1** | 0 | 0 | 0 | 1 | nc |
|  | Somewhat | 2 | **0** | 0 | 0 | 2 |  |
|  | Quite | 0 | 0 | **0** | 0 | 0 |  |
|  | Completely | 0 | 0 | 0 | **0** | 0 |  |
|  | Total | 3 | 0 | 0 | 0 | **3** |  |

| Item | Test  Retest | Not at all | Somewhat | Quite | Completely | Total | Weighted Kappa (95% CI) |
| --- | --- | --- | --- | --- | --- | --- | --- |
| Visiting  Inter-rater | not at all | **0** | 0 | 0 | 0 | 0 | nc |
|  | Somewhat | 0 | **0** | 1 | 0 | 1 |  |
|  | Quite | 0 | 0 | **0** | 0 | 0 |  |
|  | Completely | 0 | 0 | 0 | **0** | 0 |  |
|  | Total | 0 | 0 | 1 | 0 | **1** |  |
|  | | | | | | | |
| Home  Intra-rater | not at all | **0** | 0 | 0 | 0 | 0 | nc |
|  | Somewhat | 0 | **0** | 0 | 0 | 0 |  |
|  | Quite | 0 | 0 | **0** | 0 | 0 |  |
|  | Completely | 0 | 0 | 0 | **0** | 0 |  |
|  | Total | 0 | 0 | 0 | 0 | **0** |  |
|  | | | | | | | |
| Home  Inter-rater | not at all | **1** | 0 | 0 | 0 | 1 | nc |
|  | Somewhat | 0 | 0 | 0 | 0 | 0 |  |
|  | Quite | 0 | 0 | 0 | 0 | 0 |  |
|  | Completely | 0 | 0 | 0 | 0 | 0 |  |
|  | Total | 1 | 0 | 0 | 0 | **1** |  |
|  | | | | | | | |
| Independence  Intra-rater | not at all | **0** | 0 | 0 | 0 | 0 | nc |
|  | Somewhat | 0 | **0** | 0 | 0 | 0 |  |
|  | Quite | 0 | 0 | **0** | 0 | 0 |  |
|  | Completely | 0 | 0 | 1 | **0** | 1 |  |
|  | Total | 0 | 0 | 1 | 0 | 1 |  |
|  | | | | | | | |
| Independence  Inter-rater | not at all | **1** | 0 | 0 | 0 | 1 | nc |
|  | Somewhat | 1 | **0** | 0 | 1 | 2 |  |
|  | Quite | 2 | 0 | **0** | 1 | 3 |  |
|  | Completely | 0 | 0 | 0 | **0** | 0 |  |
|  | Total | 4 | 0 | 0 | 2 | **6** |  |

**Table 3. Intra- and Inter-rater reliability weighted kappa values summarised**

| Item | Intra-rater reliability | | | | | Inter-rater reliability | | | | |
| --- | --- | --- | --- | --- | --- | --- | --- | --- | --- | --- |
|  | n | %* | Weighted Kappa  (95% CI) | K_max_ | K/ K_max_ | n | %* | Weighted Kappa (95% CI) | K_max_ | K/ K_max_ |
| Better | 22 | 45.45 | 0.59 (0.21;0.98) | 0.70 | 0.85 | 29 | 55.17 | 0.43 (0.05;0.82) | 0.73 | 0.60 |
| Energy | 22 | 50.00 | 0.47 (0.05;0.89) | 0.98 | 0.48 | 19 | 36.84 | 0.44 (0.06;0.82) | 0.78 | 0.57 |
| Shortness of breath | 13 | 53.85 | 0.34 (0;0.78) | 0.76 | 0.45 | 12 | 58.33 | 0.63 (0.03;1) | 0.72 | 0.87 |
| Walking | 13 | 38.46 | 0.04 (nc) | 0.92 | 0.04 | 12 | 33.33 | 0.45 (0.04;0.87) | 0.80 | 0.56 |
| Controlling disease | 12 | 58.33 | 0.36 (0;0.88) | 0.85 | 0.43 | 17 | 52.94 | 0.74 (0.59;0.90) | 0.88 | 0.84 |
| Alive | 13 | 69.23 | 0.57 (0.17;0.97) | 0.63 | 0.91 | 15 | 53.33 | 0.38 (0.11;0.65) | 0.38 | 1 |

*% = percentage of agreement, K= kappa K_max_=maximum attainable weighted kappa CI= Confidence interval nc= not calculated
