# Supplementary material for: Reliability and validity of the Patient Benefit Assessment Scale for Hospitalised Older Patients (P-BAS HOP)
Source: BMC Geriatr. 2021 Mar 1;21:149. doi: 10.1186/s12877-021-02079-z (PMC7923656; doi:10.1186/s12877-021-02079-z)
Supplement: Supplementary file 5 — Additional file 5. [file 12877_2021_2079_MOESM5_ESM.docx]

**Additional file 5. Crosstabulations hypotheses baseline validity**

**Reliability and validity of the Patient Benefit Assessment Scale for Hospitalised Older Patients (P-BAS HOP)**

**Authors:**

1. Maria Johanna van der Kluit, MSc RN (Corresponding author)

University of Groningen, University Medical Center Groningen, University Center for Geriatric Medicine, Hanzeplein 1, 9700 RB Groningen, The Netherlands

[m.j.van.der.kluit@umcg.nl](mailto:m.j.van.der.kluit@umcg.nl)

+31503613921

1. Geke J. Dijkstra, PhD

University of Groningen, University Medical Center Groningen, Department of Health Sciences, Applied Health Research, Groningen, The Netherlands

NHL Stenden University of Applied Sciences, Research Group Living, Wellbeing and Care for Older People, Leeuwarden, The Netherlands

[g.j.dijkstra@umcg.nl](mailto:g.j.dijkstra@umcg.nl)

1. Sophia E. de Rooij, MD PhD

University of Groningen, University Medical Center Groningen, University Center for Geriatric Medicine, Groningen, The Netherlands

Medical Spectrum Twente, Medical School Twente, Enschede, The Netherlands

sejaderooij@gmail.com

**Additional file 5. Crosstabulations hypotheses baseline validity**

| Hypothesis | Answer P-BAS HOP | Answer VMS and/or RSCL | | Total | Cramér’s V |
| --- | --- | --- | --- | --- | --- |
|  |  | n (% within VMS and/or RSCL) | | |  |
|  |  | no | yes |  |  |
| Participants who indicated a lack of appetite on the VMS and/or the RSCL, are expected to have a higher priority on the goal appetite. | Doesn’t apply/ not at all important | 301 (98) | 88 (62) | 389 (86) | .50 |
|  | Somewhat important | 1 (0) | 8 (6) | 9 (2) |  |
|  | Quite important | 3 (1) | 17 (12) | 20 (4) |  |
|  | Very important | 2 (1) | 30 (21) | 32 (7) |  |
|  | Total | 307 | 143 | 450 |  |
|  | | | | | |
|  | Answer P-BAS HOP | Answer RSCL | | Total | Cramér’s V |
|  |  | n (% within RSCL) | | |  |
|  |  | no | yes |  |  |
| Participants who indicated tiredness and/ or lack of energy on the RSCL, are expected to have a higher priority on the goal energy. | Doesn’t apply/ not at all important | 122 (62) | 73 (30) | 195 (44) | .34 |
|  | Somewhat important | 5 (3) | 10 (4) | 15 (3) |  |
|  | Quite important | 30 (15) | 45 (18) | 75 (17) |  |
|  | Very important | 40 (20) | 117 (48) | 157 (36) |  |
|  | Total | 197 | 245 | 442 |  |
|  | | | | | |
|  | Answer P-BAS HOP | Answer RSCL | | Total | Cramér’s V |
|  |  | n (% within RSCL) | | |  |
|  |  | no | yes |  |  |
| Participants who indicated diarrhoea and/ or constipation on the RSCL, are expected to have a higher priority on the goal bowel movements.* | Doesn’t apply/ not at all important | 344 (91) | 3 (52) | 377 (86) | .40 |
|  | Somewhat important | 3(1) | 1 (2) | 4 (1) |  |
|  | Quite important | 11 (3) | 10 (15) | 21 (5) |  |
|  | Very important | 19 (5) | 20 (31) | 39 (9) |  |
|  | Total | 377 | 64 | 441 |  |
|  | | | | | |
|  | Answer P-BAS HOP | Answer RSCL | | Total | Cramér’s V |
|  |  | n (% within RSCL) | | |  |
|  |  | no | yes |  |  |
| Participants who indicated shortness of breath on the RSCL, are expected to have a higher priority on the goal reducing shortness of breath. | Doesn’t apply/ not at all important | 212 (86) | 54 (28) | 266 (61) | .60 |
|  | Somewhat important | 8 (3) | 6 (3) | 14 (3) |  |
|  | Quite important | 10 (4) | 34 (18) | 44 (10) |  |
|  | Very important | 18 (7) | 98 (51) | 116 (26) |  |
|  | Total | 248 | 192 | 440 |  |
|  | | | | | |
|  | Answer P-BAS HOP | Admission type | | Total | Cramér’s V |
|  |  | n (% within admission type) | | |  |
|  |  | Not acute or diagnostic | Acute or diagnostic |  |  |
| Participants who had an acute admission and/ or a diagnostic admission reason, are expected to have a higher priority on the goal wanting to know what is wrong. | Doesn’t apply/ not at all important | 143 (89) | 184 (68) | 326 (76) | .25 |
|  | Somewhat important | 2 (1) | 6 (2) | 8 (2) |  |
|  | Quite important | 5 (3) | 15 (6) | 20 (5) |  |
|  | Very important | 10 (6) | 67 (25) | 77 (18) |  |
|  | Total | 160 | 271 | 431 |  |

* To fit the assumptions of the Cramér’s V statistic, the categories somewhat and quite important were combined.
