# Supplementary material for: Reliability and validity of the Patient Benefit Assessment Scale for Hospitalised Older Patients (P-BAS HOP)
Source: BMC Geriatr. 2021 Mar 1;21:149. doi: 10.1186/s12877-021-02079-z (PMC7923656; doi:10.1186/s12877-021-02079-z)
Supplement: Supplementary file 6 — Additional file 6. [file 12877_2021_2079_MOESM6_ESM.docx]

**Additional file 6. Crosstabulations hypotheses follow-up validity**

**Reliability and validity of the Patient Benefit Assessment Scale for Hospitalised Older Patients (P-BAS HOP)**

**Authors:**

1. Maria Johanna van der Kluit, MSc RN (Corresponding author)

University of Groningen, University Medical Center Groningen, University Center for Geriatric Medicine, Hanzeplein 1, 9700 RB Groningen, The Netherlands

[m.j.van.der.kluit@umcg.nl](mailto:m.j.van.der.kluit@umcg.nl)

+31503613921

1. Geke J. Dijkstra, PhD

University of Groningen, University Medical Center Groningen, Department of Health Sciences, Applied Health Research, Groningen, The Netherlands

NHL Stenden University of Applied Sciences, Research Group Living, Wellbeing and Care for Older People, Leeuwarden, The Netherlands

[g.j.dijkstra@umcg.nl](mailto:g.j.dijkstra@umcg.nl)

1. Sophia E. de Rooij, MD PhD

University of Groningen, University Medical Center Groningen, University Center for Geriatric Medicine, Groningen, The Netherlands

Medical Spectrum Twente, Medical School Twente, Enschede, The Netherlands

sejaderooij@gmail.com

**Additional file 6. Crosstabulations hypotheses follow-up validity**

| Hypothesis | Answer P-BAS HOP | Change on Katz-15 and/or EQ-5D | | Total | Cramér’s V |
| --- | --- | --- | --- | --- | --- |
|  |  | n (% within Katz-15 and/or EQ-5D) | | |  |
|  |  | Deteriorated | Equal/ improved |  |  |
| Participants who indicated a deterioration on the Katz-15 items bathing and/ or getting dressed and/ or the EQ-5D item self-care, are expected to have a lower score on the item wash and dress yourself. | Not at all | 3 (75) | 7 (24) | 10 (30) | n.c. |
|  | Somewhat | 0 (0) | 4 (14) | 4 (12) |  |
|  | Quite | 1 (25) | 2 (7) | 3 (9) |  |
|  | Completely | 0 (0) | 16 (55) | 16 (49) |  |
|  | Total | 4 | 29 | 33 |  |
|  | | | | | |
|  | Answer P-BAS HOP | Change on Katz-15 and/or EQ-5D | | Total | Cramér’s V |
|  |  | n (% within Katz-15 and/or EQ-5D) | | |  |
|  |  | Deteriorated | Equal/ improved |  |  |
| Participants who indicated a deterioration on the Katz-15 item walking and/ or the EQ-5D item walking, are expected to have a lower score on the item walking.* | Not at all | 5 (31) | 43 (43) | 48 (41) | .23^$^ |
|  | Somewhat | 1 (6) | 21 (21) | 22 (19) |  |
|  | Quite | 3 (19) | 19 (19) | 22 (19) |  |
|  | Completely | 7 (44) | 17 (17) | 24 (21) |  |
|  | Total | 17 | 100 | 116 |  |
|  | | | | | |
|  | Answer P-BAS HOP | Change on Katz-15 | | Total | Cramér’s V |
|  |  | n (%within Katz-15) | | |  |
|  |  | Deteriorated | Equal/ improved |  |  |
| Participants who indicated a deterioration on the Katz-15 item travelling, are expected to have a lower score on the item driving. | Not at all | 3 (100) | 12 (35) | 15 (41) | n.c. |
|  | Somewhat | 0 (0) | 2 (6) | 2 (5) |  |
|  | Quite | 0 (0) | 2 (6) | 2 (5) |  |
|  | Completely | 0 (0) | 18 (53) | 18 (49) |  |
|  | Total | 3 | 34 | 37 |  |
|  | | | | | |
|  | Answer P-BAS HOP | Change on Katz-15 | | Total | Cramér’s V |
|  |  | n (%within Katz-15) | | |  |
|  |  | Deteriorated | Equal/ improved |  |  |
| Participants who indicated a deterioration on the Katz-15 item shopping, are expected to have a lower score on the item groceries. | Not at all | 3 (75) | 10 (30) | 13 (35) | n.c. |
|  | Somewhat | 0 (0) | 4 (12) | 4 (11) |  |
|  | Quite | 0 (0) | 6 (18) | 6 (16) |  |
|  | Completely | 1 (25) | 13 (39) | 14 (38) |  |
|  | Total | 4 | 33 | 37 |  |
|  | | | | | |
|  | Answer P-BAS HOP | Change on EQ-5D | | Total | Cramér’s V |
|  |  | n (% within EQ-5D) | | |  |
|  |  | Deteriorated | Equal/ improved |  |  |
| Participants who indicated a deterioration on the EQ-5D item pain/ discomfort, are expected to have a lower score on the item pain.* | Not at all | 3 (16) | 25 (30) | 28 (28) | .14^$^ |
|  | Somewhat | 5 (26) | 13 (16) | 18 (18) |  |
|  | Quite | 2 (11) | 17 (21) | 19 (19) |  |
|  | Completely | 9 (47) | 28 (34) | 37 (36) |  |
|  | Total | 19 | 83 | 102 |  |
|  | | | | | |
| Hypothesis | Answer P-BAS HOP |  | | Total | Cramér’s V |
|  |  | n (% within VMS) | | |  |
|  |  | yes | no |  |  |
| Participants who indicated a lack of appetite on the VMS, are expected to have a lower score on the item appetite.* | Not at all | 8 (42) | 7 (27) | 15 (33) | .46 |
|  | Somewhat | 5 (26) | 2 (8) | 7 (16) |  |
|  | Quite | 4 (21) | 3 (12) | 7 (16) |  |
|  | Completely | 2 (11) | 14 (54) | 16 (36) |  |
|  | Total | 19 | 26 | 45 |  |
|  | | | | | |
|  | Answer P-BAS HOP | Change on MSPP | | Total | Cramér’s V |
|  |  | n (% within MSPP) | | |  |
|  |  | Deteriorated | Equal/ improved |  |  |
| Participants who indicated a deterioration on the MSPP item organised sports and/ or the MSPP item done something with others that required considerable physical effort, are expected to have a lower score on the item sports. | Not at all | 3 (50) | 6 (40) | 9 (43) | n.c. |
|  | Somewhat | 1 (17) | 2 (13) | 3 (14) |  |
|  | Quite | 0 (0) | 6 (40) | 6 (29) |  |
|  | Completely | 2 (33) | 1 (7) | 3 (14) |  |
|  | Total | 6 | 15 | 21 |  |
|  | Answer P-BAS HOP | Change of MSPP or SF-36 social functioning | | Total | Cramér’s V |
|  |  | n (% within MSPP or SF-36) | | |  |
|  |  | Deteriorated | Equal/ improved |  |  |
| Participants who indicated a deterioration on the MSPP item seeing family/ acquaintances or the question ‘During the past 4 weeks, how much of the time has your physical health or emotional problems interfered with your social activities?’, are expected to have a lower score on the item visiting family or friends. | Not at all | 3 (50) | 13 (54) | 16 (53) | n.c. |
|  | Somewhat | 1 (17) | 3 (13) | 4 (13) |  |
|  | Quite | 0 (0) | 2 (8) | 2 (7) |  |
|  | Completely | 2 (33) | 6 (25) | 8 (27) |  |
|  | Total | 6 | 24 | 30 |  |
|  | | | | | |
|  | Answer P-BAS HOP | Change in living situation | | Total | Cramér’s V |
|  |  | n (% within living situation) | | |  |
|  |  | To sheltered/ nursing home | Equal/ improved |  |  |
| Participants who moved from independent living to sheltered living or a nursing home, are expected to score lower on the item back to own home. | Not at all | 1 (50) | 0 (0) | 1 (9) | n.c. |
|  | Somewhat | 0 (0) | 1 (11) | 1 (9) |  |
|  | Quite | 0 (0) | 0 (0) | 0 (0) |  |
|  | Completely | 1 (50) | 8 (89) | 9 (82) |  |
|  | Total | 2 | 9 | 11 |  |

^$^ Association is reversed to hypothesis

* To fit the assumptions of the Cramér’s V statistic, the categories somewhat and quite were combined.
